# Multidimensional body image and self-esteem: a latent profile analysis differentiating orthorexia nervosa and exercise addiction from disordered eating

**DOI:** 10.1186/s12888-026-08219-2

**Published:** 2026-05-25

**Authors:** Hanna Wachten, Jana Strahler

**Affiliations:** 1 Department of Sport and Sport Science, Albert-Ludwigs-University Freiburg, Sportpsychology, Freiburg, Germany; 2 Department of Psychotherapy and System Neuroscience, Justus Liebig University Giessen, Giessen, Germany

**Keywords:** Disordered eating, Orthorexia nervosa, Exercise addiction, Multidimensional body image, Self-esteem, Latent profile analysis

## Abstract

**Background:**

Orthorexia Nervosa (ON), the obsessive preoccupation with healthy eating, and Exercise Addiction (EA), the behavioral addiction to exercise, are controversially discussed as potential mental disorders. The unclear distinction between these conditions and disordered eating, especially regarding restrictive eating and instrumental exercise behaviors, contributes to this debate. The objective of the study was to ascertain whether latent profiles can be identified that support the independence of ON and EA from disordered eating. It was hypothesized that ON profiles would be characterized by stronger health orientation, EA profiles by greater fitness orientation, and disordered eating profiles by heightened preoccupation with overweight, appearance concerns, and lower self-esteem.

**Methods:**

A total of 661 participants (77.2% women, age *M* ± *SD* = 26.74 ± 9.68) completed an online survey including the Düsseldorf Orthorexia Scale, Revised Exercise Addiction Inventory, Eating Disorder Examination-Questionnaire, Multidimensional Body Self-Relations Questionnaire, and Rosenberg Self-Esteem Scale. Latent profile analysis was used to identify distinct subgroups of ON, EA, and disordered eating, which were subsequently compared on self-esteem and body image measures using one-way ANOVAs.

**Results:**

The latent profile analysis yielded five distinct profiles: In addition to two “healthy” clusters – one characterized by non-pathological eating behaviors and the other by low commitment to both diet and exercise – three profiles were identified as being of potential clinical interest. These included: (I) disordered eating, ON and instrumental exercise; (II) disordered eating alone; and (III) EA. Notably, no profile was characterized exclusively by ON. Profile I – which exhibited the highest levels of ON, EA and disordered eating – was marked by the lowest self-esteem and the strongest concerns about appearance, overweight, fitness, and health. Conversely, profile III was characterized by only modestly elevated EA levels, favorable body image, and self-esteem.

**Conclusions:**

These findings question the independence of ON, as it was closely intertwined with disordered eating pathology rather than forming a distinct behavioral pattern. Exercise addiction showed relatively low symptom severity in the absence of disordered eating, highlighting the risk of falsely identifying instrumental exercise as EA. Instrumental exercise and orthorexic tendencies may worsen negative body image in disordered eating by increasing pressure on appearance, fitness, and health of individuals.

**Clinical trial number:**

Not applicable.

**Supplementary Information:**

The online version contains supplementary material available at 10.1186/s12888-026-08219-2.

## Background

Whether *Orthorexia Nervosa* (ON) and *Exercise Addiction* (EA) represent distinct clinical entities or rather reflect specific constellations of disordered eating related symptoms remains a topic of ongoing debate [1, 2]. Although they have gained increased attention in recent years, neither ON nor EA are currently recognized as formal diagnoses in classification systems such as DSM-5 [3] or ICD-11 [4]. The lack of formal recognition is not merely a technical gap but reflects the conceptual uncertainty regarding whether ON and EA represent distinct clinical entities or rather overlap with dimensions of disordered eating, particularly those related to weight and shape concerns.

According to expert consensus [5, 6], ON refers to an obsessive preoccupation with “correct” or “healthy” eating, characterized by rigid and exaggerated beliefs about the health effects of certain foods. This preoccupation is accompanied by excessive time and effort devoted to planning, sourcing, preparing, and consuming food. Deviating from self-imposed dietary rules results in significant psychological distress. Over time, the range of permitted foods may narrow, sometimes leading to malnutrition and underweight - an outcome that stands in contrast to the original intent of promoting health. EA, on the other hand, has been described in proposed criteria [7, 8] as a potential behavioral addiction characterized by the loss of control over exercise behavior. Prioritizing exercise over other life domains is theoretically conceptualized as impairing psychosocial functioning, including social or occupational conflicts. Affected individuals use exercise as a primary strategy for mood regulation and appear to lack alternative coping strategies. Consequently, they experience withdrawal symptoms when unable to exercise. Over time, tolerance develops, requiring progressively greater amounts or intensity of exercise to achieve the same mood-regulating effect. Attempts to reduce exercise fail, even when individuals are aware of harmful consequences such as injury or exhaustion.

Prevalence estimates for ON and EA vary widely depending on the assessment instruments and study populations. For ON, studies using non-validated measures have reported prevalence rates as high as 90.6%, whereas estimates based on more robust instruments range from 2.5% to 10.5%, with slightly higher rates observed in women than in men [9, 10]. Estimates for EA range from 3.0% to 15.4%, with inconsistent findings regarding gender differences [11, 12]. However, these figures are likely inflated due to false-positive classifications [11, 13]. In comparison, Anorexia Nervosa (AN) and Bulimia Nervosa (BN) affect fewer than 1–3% of women and below 1% of men, whereas disordered eating behaviors are observed in about 10% of individuals [14, 15].

ON and EA are moderately intercorrelated [16] and share symptom-level similarities with eating disorders, particularly in terms of rigid dietary and exercise behaviors [17, 18]. Across eating disorders, excessive exercise is common but unevenly distributed, with approximately 48% of patients engaging in it to control weight or shape – most prominently in AN and BN, less so in other specified feeding or eating disorders, and comparatively rarely in binge-eating disorder [19]. In contrast, EA is conceptualized as being primarily driven by exercise for its own sake rather than instrumentally for appearance-related reasons [11]. Similarly, ON has been proposed to differ from other eating disorders, particularly those involving restrictive eating patterns, in that food avoidance is primarily motivated by perceived long-term health consequences. In contrast, restriction in AN and BN is typically driven by weight and shape concerns, whereas in avoidant/restrictive food intake disorder it is related to the avoidance of immediate adverse consequences (e.g., choking) or aversive sensory experiences [5, 20, 21]. However, empirical findings challenge this assumption, indicating that weight control motives may often supersede health as the underlying driver of orthorexic eating behavior [22, 23]. Moreover, meta-analytic evidence suggests substantial overlap with disordered eating symptoms, both for ON (*r* = 0.36) and for EA (*OR* = 3.71) [24, 25]. Thus, it remains unclear whether ON and EA reflect distinct constructs or rather overlapping symptoms within the broader spectrum of disordered eating.

Research on body image in eating disorders and disordered eating has traditionally focused on appearance-related evaluations, distortions, and dissatisfaction. The overvaluation of appearance and its negative evaluation contribute to and maintain low self-esteem, which in turn is associated with dysfunctional body-related behaviors such as restrictive eating or compulsive exercise [26, 27]. The role of self-esteem in ON and EA remains underexplored, although initial evidence suggests that lower self-esteem may contribute to the rigid eating and exercise patterns characteristic of ON and EA [28–31]. Previous studies on body image in these conditions have primarily focused on appearance dissatisfaction, with inconsistent findings for ON and moderate associations for EA [20, 31–34].

Beyond appearance, body image is increasingly understood to be multidimensional, including both evaluation and orientation toward one’s appearance, health, and fitness. Evaluation refers to an individual’s level of satisfaction, while orientation denotes the cognitive and behavioral preoccupation with these aspects, including their perceived significance to the individual [35, 36]. Individuals with eating disorders typically exhibit lower appearance satisfaction, but greater appearance orientation, and heightened overweight preoccupation compared to healthy controls [37, 38]. This broader perspective may offer valuable insights into the body image profiles of ON and EA. Since appearance-based weight control is not considered the primary motive in ON and EA, health and fitness should be the central aspects of body image in these conditions. Pauzé et al. [39] reported moderate to strong associations of ON, not only with fitness and health orientation, but with overweight preoccupation and appearance orientation as well. Using hierarchical cluster analysis, Yakın et al. [40] identified a distinct ON cluster showing intermediate levels of appearance orientation and overweight preoccupation – lower than in disordered eating, but higher than in healthy individuals. Aligning with the concept of ON, this cluster had the highest levels of health and fitness orientation. Aside from these exceptions, previous studies examining multidimensional body image in ON have relied on psychometrically inadequate instruments to assess orthorexic tendencies [41–45]. Furthermore, there is a lack of empirical evidence on the multidimensional body image profile associated with EA to date. Adopting this broader perspective on body image may help clarify the unique profiles and clinical descriptions of ON and EA, yet previous studies have only partially addressed this issue.

To address the knowledge gaps described, the present study aimed to examine whether distinct latent profiles of ON and EA can be identified that are not accompanied by elevated appearance-focused disordered eating symptoms. These profiles were then compared on multidimensional body image and self-esteem to investigate how ON and EA differ from disordered eating in their patterns. We hypothesized that profiles high in ON but low in disordered eating would show stronger health orientation, whereas EA-dominated profiles would display a higher fitness orientation. In contrast, profiles characterized by elevated disordered eating but low ON and EA were expected to show greater appearance orientation, lower appearance evaluation, increased overweight preoccupation, and reduced self-esteem.

## Methods

### Participants and data collection

Participants were recruited online (www.soscisurvey.de) using convenience sampling through various channels, i.e. university mailing lists, social media, and flyers. The study was advertised as “Nutrition, Emotion, and Attention”. Participation was voluntary and incentivized by entry into a prize draw for ten €20 vouchers. Completion-time checks were performed using the Relative Speed Index, with values above 2 indicating unusually short completion times [46]. Apart from one excluded participant, open-ended responses suggested plausible data quality for both short and long completion times (*M* ± *SD =* 32.76 ± 9.93). Inclusion criteria were the minimum age of 18 and fluency in German. After excluding two underage participants, the final sample consisted of 661 participants. The data was previously analyzed in the context of exercise activities separated by sports categories associated with ON and EA [47].

### Measures

Participants provided sociodemographic data including gender (female, male, diverse), age, body mass index (BMI), school education (*none*, *school-leaving certificate*, *intermediate school-leaving certificate*, *qualification for university entrance*), and employment (*(self)-employed*,* unemployed*,* retired*,* in vocational training*,* studying).* Participants reported current (i.e., within last 12 weeks) in- or outpatient psychotherapy treatment and past (i.e., before the last 12 weeks) psychotherapy treatment. Participants self-reported current (within past three months) and past (from the age of ten until three months ago) eating disorder diagnoses using two single-choice items. For both items, participants indicated whether health care specialists had diagnosed them with *Anorexia nervosa*, *Bulimia nervosa*, *Binge Eating Disorder*, or *Eating Disorder Not Otherwise Specified*. Moreover, exercise activity (minutes per week) was measured by the pertinent subscale of the Physical Activity, Exercise, and Sport Questionnaire ([Bewegungs- und Sportaktivität Fragebogen] BSA-F;. Participants indicated the frequency and duration of exercise sessions from up to three sports. We excluded self-reported physical activities that do not qualify as exercise (e.g. walking the dog), i.e. activities that were not planned or structured with the aim of improving fitness [48]. We then computed an overall exercise score using the fitevalapp [49].

The Düsseldorf Orthorexia Scale (DOS), [50] measured orthorexia nervosa. The ten items use a 4-point Likert scale, ranging from 1 to 4, with higher sum scores indicating more pronounced orthorexic behavior (range: 10 to 40). A preliminary cut-off ≥ 30 indicates a significant risk for ON. The DOS is a validated tool for assessing ON; however, its one-dimensional factor structure has been debated, with some studies identifying three- and five-factor structures [51, 52]. Internal consistency was Cronbach’s α = 0.87 in the present sample.

The Revised Exercise Addiction Inventory (EAI-R), [53] assessed exercise addiction. The six items use a 6-point Likert scale, ranging from 1 to 6. Each item assesses one component of behavioral addictions: salience, mood modification, tolerance, withdrawal symptoms, conflict, and relapse [54]. Higher sum scores (range: 6 to 36) represent greater levels of EA. Sum scores ≥ 29 indicate a significant risk for EA, based on the approximation (the upper 15% of scale values) off the EAI [55]. Internal consistency was Cronbach’s α = 0.87 in the present sample.

The Short Eating Disorder Examination-Questionnaire (EDE-Q8), [56, 57] measured disordered eating behavior. The eight items refer to the past 28 days on 7-point scales, ranging from 0 to 6. Higher mean scores (range: 0 to 6) indicate greater eating psychopathology. Internal consistency was Cronbach’s α = 0.92 in the present sample.

The Multidimensional Body-Self Relations Questionnaire (MBSRQ), [58, 59] assessed self-attitudinal aspects of body-image. The 71 items use 5-point Likert scales, ranging from 1 to 5. In addition to the seven mean scores for appearance evaluation, appearance orientation, fitness evaluation, fitness orientation, health evaluation, health orientation, and illness orientation (i.e., alertness or reactivity to symptoms of illness), another three mean score scales can be computed (range: 1–5): Self-classified weight reflects how individuals subjectively rate their own body weight, ranging from very underweight to very overweight. Overweight preoccupation reflects fat anxiety, weight vigilance, weight loss dieting and restraint eating. The body areas satisfaction scale, which assesses satisfaction with specific body parts and overall appearance, was not analyzed in this study, because it is highly overlapping with appearance evaluation, *r* = 0.842, *p* < .001. In this sample, internal consistencies of scales ranged from Cronbach’s α = 0.70 to 0.92.

The Rosenberg Self-Esteem Scale (RSES), [60, 61] assessed global self-esteem as a measure of self-worth and self-acceptance. The ten items are rated on a 4-point Likert scale ranging from 0 to 3. Higher sum scores (range: 0 to 30) indicate greater levels of self-esteem, with sum scores < 15 indicating low, 15–25 normal, and ≥ 25 high self-esteem. In this sample, internal consistency was Cronbach’s α = 0.92.

### Statistical analyses

All analyses were conducted using R v4.2.0 [62]. Descriptive statistics were computed for sociodemographic data, psychotherapy treatment, current and past eating disorders, DOS, EAI-R, and EDE-Q8 (*M* ± *SD*, min, and max for metric variables, *N* and % for categorial variables). To preliminarily analyze the conceptual discrimination of Exercise Addiction, orthorexic eating behaviors, and disordered eating, we performed a confirmatory factor analysis with the six EAI-R items, ten DOS items, and eight EDE-Q8 items in a three-factor model. Due to multivariate non-normality, Zhou-Shao’s Test: *p* < 0.001 (*mvnormalTest* package), [63, 64], and ordinal data with 4-point to 7-point Likert scales, we used Weighted Least Squares Mean and Variance Adjusted estimation [65, 66]. Acceptable model fit was indicated by the following fit indices [67]: Root mean squared error of approximation (RMSEA < 0.07, 90%-CI = ~ 0 to < 0.08), comparative fit index (CFI > 0.95), standardized root mean squared residual (good SRMR < 0.05, acceptable SRMR < 0.08). We further explored the latent factor structure in exploratory factor analyses using Weighted Least Squares estimation, oblimin rotation and polychoric correlation matrix. Subsequently, we performed a latent profile analysis with the scales DOS, EAI-R, and EDE-Q8 following recommendations of Spur et al. [68] and using the tidyLPA package [69]. Sample size was sufficient to accurately identify profile clusters according to rule of thumb *N* ≥ 500 [70]. No data were missing. Due to univariate (all *p* < .001) and multivariate non-normality (Zhou-Shao’s Test: *p* = 0.028), we used Maximum-Likelihood estimation with robust standard errors. We compared the fit of one to six profiles for all Mclust model specifications. The optimal number of profiles was determined using the Bootstrapped Likelihood Ratio test [71] and an analytic hierarchy process [72] with the following fit indices (with lower values indicating a better fit): Akaike’s Information Criterion (AIC), Approximate Weight of Evidence (AWE), Bayesian Information Criterion (BIC), Classification Likelihood Criterion (CLC), and Kullback Information Criterion (KIC). We further report entropy and classification probabilities of profiles. Interpretability was carefully evaluated using z-standardized mean values, considering the theoretical framework of ON, EA, and disordered eating. Resulting profiles were compared on DOS, EAI-R and EDE-Q8. Given the unequal sample sizes, we used Welch-ANOVAs when data was normal but heteroskedastic, Kruskal-Wallis tests when both normality and homoskedasticity assumptions were violated, and one-way ANOVAs in other cases [73, 74]. Criterion validity was established using descriptive comparisons on BMI, self-reported eating disorder diagnoses, and exercise activity. Subsequently, main effects of profile were tested for all MBSRQ scales and RSES. Games-Howell-Post-Hoc-Tests were computed, reported as 95% confidence intervals for mean differences, since they perform well with non-balanced sample sizes, non-normality and heteroskedasticity and adjust for multiple testing [75].

## Results

### Sample description

The final sample (*N* = 661) comprised 77.2% women (*N* = 510), 22.4% men (*N* = 148), and 0.6% participants who indicated their gender as diverse (*N* = 3). Regarding school education, 631 participants had general qualifications for university entrance (95.5%), 27 participants had intermediate school-leaving certificates (4.1%), and two participants had school-leaving certificates (0.3%). Of all participants, 32.4% were working or self-employed (*N* = 214), 1.2% unemployed (*N* = 8), 1.7% retired (*N* = 11), 2.1% in vocational training (*N* = 14), and 62.6% studying (*N* = 414). Eighty-six participants reported currently receiving outpatient psychotherapy treatment (13.0%), 13 reported inpatient psychotherapy treatment (2.0%), and 129 participants reported having received psychotherapy in the past (19.5%), while 460 participants reported never having received psychotherapy (69.6%) and 560 participants that they had never been diagnosed with any eating disorder (84.7%). Further descriptive statistics are provided in Table 1.

**Table 1:** Descriptive statistics M ± SD (Min, Max) or N (%) for all participants and per profile

	Profiles					Total Sample
	DisOrEx	DisEat	AddEx	NonPath	LowCom	
*N* women men diverse	105 97 (92.4%) 8 (7.6%) -	131 113 (86.3%) 17 (13.0%) 1 (0.8%)	231 162 (70.1%) 68 (29.4%) 1 (0.4%)	147 103 (70.1%) 43 (29.3%) 1 (0.7%)	47 35 (74.5%) 12 (25.5%) -	661 510 (77.2%) 148 (22.4%) 3 (0.5%)
age	26.11 ± 7.11 (18, 60)	28.64 ± 12.26 (18, 70)	26.40 ± 9.70 (18, 72)	25.20 ± 7.43 (19, 68)	29.28 ± 11.52 (18, 56)	26.74 ± 9.68 (18, 72)
exercise	213.85 ± 245.22 (0, 1560)	118.69 ± 183.30 (0, 1050)	253.28 ± 248.15 (0, 1320)	207.93 ± 214.87 (0, 1440)	54.39 ± 102.94 (0, 562.50)	196.13 ± 228.82 (0, 1560)
BMI	21.25 ± 3.03 (13.82, 29.02)	24.83 ± 5.38 (17.21, 60.09)	22.22 ± 3.18 (14.71, 41.66)	21.27 ± 2.74 (15.78, 31.25)	23.67 ± 4.85 (17.85, 37,42)	22.48 ± 3.97 (13.82, 60.09)
< 18.5 18.5 < 25 25 < 30 ≥ 30	17 (16.2%) 76 (72.4%) 12 (11.4%) –	3 (2.3%) 73 (55.7%) 40 (30.5%) 15 (11.5%)	17 (7.4%) 172 (74.5%) 38 (16.5%) 4 (1.7%)	21 (14.3%) 16 (10.9%) 108 (73.5%) 1 (0.7%)	2 (4.3%) 32 (68.1%) 8 (17.0%) 5 (10.6%)	60 (9.1%) 461 (69.7%) 114 (17.3%) 25 (3.8%)
EDE-Q8	4.44 ± 1.07 (1.30; 1.62)	3.54 ± 0.91 (2.00; 6.00)	1.34 ± 0.56 ^a^ (0.00, 2.50)	0.32 ± 0.24 (0.00, 0.75)	1.17 ± 0.69^a^ (0.00, 2.75)	2.03 ± 1.66 (0.00; 6.00)
DOS at risk (%)	30.24 ± 4.31 (22; 40) 53 (50.5%)	18.66 ± 3.59 (10; 27) –	20.35 ± 4.07 (10; 32) 3 (2.2%)	16.07 ± 2.88 (10; 23) –	12.79 ± 1.68 (10; 16) –	20.10 ± 6.12 (10; 40) 56 (8.5%)
EAI-R at risk (%)	24.35 ± 7.03 (6; 36) 33 (31.4%)	15.28 ± 5.81 ^a^ (6; 28) –	20.11 ± 5.58 (6; 36) 15 (6.5%)	16.88 ± 5.92 ^a^ (6; 35) 4 (2.7%)	20.11 ± 5.58 (6; 12) –	18.23 ± 7.10 (6; 36) 52 (7.9%)
EDD (C/P) AN BN BED EDNOS	40 (38.1%) / 50 (47.6%) 18 (17.1%) / 22 (21.0%) 5 (7.8%) / 10 (9.5%) 4 (3.8%) / – 13 (12.4%) / 18 (17.1%)	7 (5.3%) / 15 (11.5%) 1 (0.8%) / 4 (3.1%) 2 (1.5%) / 6 (4.6%) 3 (2.3%) / – 1 (0.8%) / 5 (3.8%)	5 (2.2%) / 21 (9.1%) – / 10 (4.3%) 2 (0.9%) / 3 (1.3%) – / 1 (0.4%) 3 (1.3%) / 7 (3.0%)	– / 3 (2.9%) – / – – / 2 (1.4%) – / – – / 1 (0.7%)	– / 3 (6.4%) – / – – / 1 (2.1%) – / – – / 2 (4.3%)	52 (7.9%) / 92 (13.9%) 19 (2.9%) / 36 (5.5%) 9 (1.4%) / 22 (3.3%) 7 (10.6%) / 1 (0.2%) 17 (2.6%) / 33 (5.0%)
MBSRQ						
AO	3.69 ± 0.62 ^a^ (2.46, 4.85)	3.50 ± 0.69 ^a, b^ (1.15, 4.92)	3.33 ± 0.58 ^b^ (1.85, 4.92)	3.05 ± 0.64 ^c^ (0.57, 1.08)	2.96 ± 0.68 ^c^ (1.54, 4.08)	3.33 ± 0.67 (1.08, 4.92)
AE	2.64 ± 0.9 ^a^ (1.00, 5.00)	2.95 ± 0.79 ^a^ (1.00, 5.00)	3.77 ± 0.59 ^b^ (2.14, 5.00)	3.94 ± 0.60 ^b^ (1.57, 5.00)	3.33 ± 0.73 (1.43, 4.86)	3.43 ± 0.86 (1.00, 5.00)
OWP	3.85 ± 0.79 (1.00, 5.00)	3.11 ± 0.79 (1.00, 5.00)	2.23 ± 0.71 (1.00, 4.00)	1.53 ± 0.52 ^a^ (1.00, 3.25)	1.69 ± 0.65 ^a^ (1.00, 3.25)	2.47 ± 1.06 (1.00, 5.00)
SCW	3.07 ± 0.73 ^a, b^ (1.00, 5.00)	3.47 ± 0.64 (2.00, 5.00)	2.95 ± 0.48 ^a, c^ (1.00, 4.00)	2.80 ± 0.39 (2.00, 4.00)	3.14 ± 0.61 ^b, c^ (2.00, 5.00)	3.05 ± 0.60 (1.00, 5.00)
FO	3.76 ± 0.74 ^a, b^ (1.38, 5.00)	3.04 ± 0.81 (1.46, 4.77)	3.83 ± 0.74 ^a^ (1.31, 5.00)	3.59 ± 0.73 ^b^ (1.46, 5.00)	2.46 ± 0.75 (1.15, 4.54)	3.51 ± 0.86 (1.00, 5.00)
FE	3.42 ± 0.76 ^a, b^ (1.40, 5.00)	3.18 ± 0.82 ^a, c^ (1.40, 4.80)	3.79 ± 0.68 ^d^ (1.40, 5.00)	3.81 ± 0.68 ^d^ (1.40, 5.00)	3.23 ± 0.84 ^b, c^ (1.00, 5.00)	3.58 ± 0.78 (1.00, 5.00)
HO	3.68 ± 0.61 ^a^ (1.88, 4.88)	3.12 ± 0.58 (1.75, 4.25)	3.58 ± 0.58 ^a^ (1.50, 4.75)	3.40 ± 0.53 (2.00, 4.62)	2.71 ± 0.61 (1.50, 4.12)	3.40 ± 0.64 (1.50, 4.88)
IO	2.98 ± 0.81 ^a, b,c^ (1.2, 4.80)	2.86 ± 0.81 ^a, d,e^ (1.20, 5.00)	2.92 ± 0.72 ^b, d,f^ (1.20, 5.00)	2.91 ± 0.67 ^c, e,f^ (1.00, 4.60)	2.31 ± 0.82 (1.00, 4.20)	2.87 ± 0.76 (1.00, 5.00)
HE	3.44 ± 0.84 ^a^ (1.00, 5.00)	3.60 ± 0.80 ^a, b^ (1.67, 5.00)	3.99 ± 0.65 ^c, d^ (1.67, 5.00)	4.06 ± 0.66 ^c, e^ (1.83, 5.00)	3.91 ± 0.68 ^b, d,e^ (1.67, 4.83)	3.84 ± 0.75 (1.00, 5.00)
RSES	14.10 ± 7.19 (0, 29)	18.11 ± 7.29 (0, 30)	22.30 ± 5.23 ^a^ (5, 30)	23.92 ± 5.18 ^b^ (6, 30)	21.62 ± 6.39 ^a, b^ (8, 30)	20.48 ± 6.96 (0, 30)
< 15 15 < 25 ≥ 25	55 (52.4%) 42 (40.0%) 8 (7.6%)	43 (32.8%) 59 (45.0%) 29 (22.1%)	18 (7.8%) 119 (51.5%) 94 (40.7%)	12 (8.2%) 51 (34.7%) 84 (57.1%)	9 (19.2%) 18 (38.3%) 20 (42.6%)	137 (20.7%) 289 (43.7%) 235 (35.6%)

Note. Exercise = Weekly exercise activity in minutes, EDE-Q8 = Short Eating Disorder Examination-Questionnaire, DOS = Düsseldorf Orthorexia Scale, EAI-R = Revised Exercise Addiction Inventory, EDD (C/P) = Self-reported Eating Disorder Diagnoses (Current / Past), AN = Anorexia nervosa, BN = Bulimia nervosa, BED = Binge Eating Disorder, EDNOS = Eating Disorder Not Otherwise Specified, MBSRQ = Multidimensional Body-Self Relations Questionnaire, AO = appearance orientation, AE = appearance evaluation, OWP = overweight preoccupation, SCW = self-classified weight, FO = fitness orientation, FE = fitness evaluation, HO = health orientation, IO = illness orientation, HE = health orientation, RSES = Rosenberg Self-Esteem Scale. – indicates *N* (%) = 0. Profiles sharing the same superscript letter do not differ significantly at *p* < 0.05 on post hoc comparisons for EDE-Q8, DOS, EAI-R, MBSRQ scales, and RSES

### Factor analysis

Preliminary confirmatory factor analysis indicated a mediocre model fit for the three-factor model of the EAI-R, DOS and EDE-Q8 items, χ²(249) = 1678.385, *p* < 0.001, RMSEA = 0.088 (90%-CI = 0.084; 0.092), CFI = 0.937, SRMR = 0.09 (see Table S1 in Supplementary Material for all parameter estimates). The strong correlation between the orthorexic and disordered eating factors, *r* = 0.616, *p* < .001, suggests a high degree of overlap in symptoms, while the Exercise Addiction and disordered eating factors were moderately related, *r* =0.495, *p* < .001. Since modification indices ≥ 10 (see Table S2) suggested several cross-factor loadings and item covariances to increase the model fit, we further explored the factor structure in exploratory factor analyses. Assumptions were met, since Measure of Sampling Adequacy = 0.92 was marvelous and Bartlett’s test significant, χ²(276) = 9756.43, *p* < .001. As displayed in Fig. S1, the scree plot and the eigenvalue > 1 criterion indicated a three-factor solution. Although Horn’s parallel analysis and Velicer’s Minimum Average Partial test suggested a four-factor model, the three-factor solution was deemed more appropriate, as the fourth factor was considered unreliable due to insufficient item loadings [76, 77]. Key parameters of both the three- and four-factor solutions are reported in Tables S3–S6, which provide detailed information for readers who wish to explore the analysis in greater depth. Items of the EAI-R had high communalities ≥ 0.40, except for item 2 (interpersonal conflicts due to the extent of exercise). This item may require further refinement or re-evaluation in future research, as its low communality suggests poor fit within the overall factor structure. DOS’s items varied in their communalities from h^2^ = 0.29 to 0.59, with the poorest fit of item 5 (positive attitude regarding the healthiness of one’s diet compared to others) and highest fit for item 8 (rumination about one’s healthy diet and its influence on daily life). The uniqueness of DOS’s items was high and ranged from u^2^ = 0.43 to 0.68 as well. EDE-Q8’s items had high communalities, while item 3 (impairment of concentration due to cognitive preoccupation with diet, foods, and calorie intake) loaded comparably onto two factors.

### Latent profile analysis

Based on the analytic hierarchy process, the latent profile analysis indicated a five-profile solution, estimated under a model with variances and covariances fixed to zero. As shown in Table 2, the five-profile solution demonstrated the most favorable fit indices, and adding a sixth profile did not yield further improvement (Bootstrap Likelihood Ratio Test, *p* = .297). Entropy indicated that cases could be assigned to the correct latent profile with acceptable certainty [78]. Classification probabilities (ranging from 0.62 to 0.92) and the reasonable sample size of the fifths profile (7.1%) also suggested that the latent profile analysis model provided a reliable fit for the data. The five-profile solution was theoretically interpretable, with each profile showing a distinct pattern across the three variables. Notably, the profiles also varied on other descriptive variables, as detailed in Table 1.

**Table 2:** Fit indices of all latent profile analysis solutions for standardized EDE-Q8, DOS, and EAI-R

Profile solutions	AIC	AWE	BIC	CLC	KIC	Entropy	Classification probability	BLRT
I	563.51	5718.43	5663.47	5626.51	5645.51	1.00	1.00–1.00	
II	5236.45	5374.58	5281.38	5218.19	5249.45	0.87	0.89–0.98	.001
III	5185.56	5379.98	5248.47	5158.97	5202.56	0.70	0.82–0.91	.001
IV	5065.23	5315.54	5146.11	5030.69	5086.23	0.73	0.75–0.93	.001
**V**	**5051.98**	**5358.27**	**5150.84**	**5009.42**	**5076.98**	**0.72**	**0.62–0.92**	**.001**
VI	5055.35	5417.76	5172.19	5004.62	5084.35	0.63	0.56–0.89	.297

Note. EDE-Q8 = Short Eating Disorder Examination-Questionnaire, DOS = Düsseldorf Orthorexia Scale, EAI-R = Revised Exercise Addiction Inventory. AIC = Akaike’s Information Criterion, AWE = Approximate Weight of Evidence, BIC = Bayesian Information Criterion, CLC = Classification Likelihood Criterion, KIC = Kullback Information Criterion, BLRT = *p*-value for the bootstrapped likelihood ratio test

**Fig. 1:**
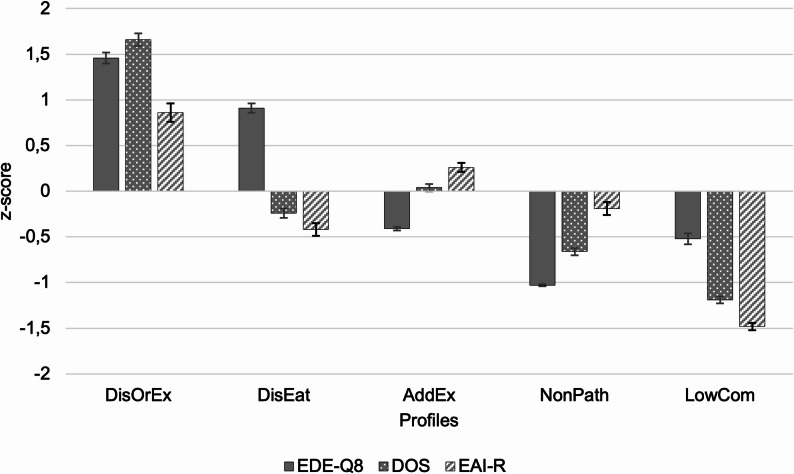
Z-standardized mean values and standard errors of EDE-Q8, DOS, and EAI-R across the five profiles. *Note*. EDE-Q8 = Short eating disorder examination-questionnaire, DOS = Düsseldorf Orthorexia Scale, EAI-R = Revised Exercise Addiction Inventory

Shown in Fig. 1, the first profile was characterized by the highest levels of EDE-Q8, DOS, and EAI-R. In this profile, 38.1% of participants reported a current eating disorder diagnosis; 50.5% were at risk for ON, and 31.4% for EA. Given the high prevalence of disordered eating symptoms, the elevated EAI-R scores in this profile likely reflect instrumental exercise, i.e. exercise performed to compensate for weight or shape concerns, rather than true Exercise Addiction [11]. Thus, we labeled the first profile *Disordered and Orthorexic Eating Behaviors with Instrumental Exercise* (DisOrEx, *N* = 105). Profile II showed elevated levels of disordered eating only and was therefore labeled *Disordered Eating* (DisEat, *N* = 131). We will refer to profile III as *Exercise Addiction* (AddEx, *N* = 231), as it was characterized by above-average EAI-R scores only. However, this elevation was modest, amounting to just 0.26 standard deviations above the sample mean. Consequently, it remains uncertain whether this profile reflects clinically meaningful manifestations of an addictive exercise pattern or rather non-clinical variations in exercise involvement. This issue is addressed in more detail in the Discussion. Profile IV showed low levels on all three variables, particularly disordered eating, and was labeled *Non-Pathological Eating* (NonPath, *N* = 147). Finally, profile V displayed low scores as well, especially on DOS and EAI-R, and was termed *Low Commitment to Diet and Exercise* (LowCom, *N* = 47), aligning with the lowest weekly exercise activity of all profiles (see Table 1). Kruskal-Wallis tests revealed significant main effects of profile classification for EDE-Q8 (χ²[4] = 475.84, *p* < .001), DOS (χ²[4] = 450.25, *p* < .001), and EAI-R (χ²[4] = 220.92, *p* < .001). Post hoc tests revealed that most profile comparisons were significant at *p* < .001, as detailed in Table S7 and indicated by superscripts in Table 1. The only non-significant differences emerged between profiles AddEx and LowCom on the EDE-Q8 (*M*_Δ_ = -0.17, 95%-CI [-0.48, 0.13]) and between profiles DisEat and NonPath on the EAI-R (*M*_Δ_ = 1.60, 95%-CI [-0.33, 3.54]).

### Comparison of profiles on measures of body image and self-esteem

Main effects of profile were significant for RSES and all MBSRQ scales and RSES (RSES: χ²[4] = 146.36, *p* < .001, appearance orientation: *F*(4, 656) = 22.40, *p* < .001, appearance evaluation: χ²[4] = 212.13, *p* < .001, overweight preoccupation: χ²[4] = 367.13, *p* < .001, self-classified weight: χ²[4] = 96.51, *p* < .001, fitness orientation: *F*(4, 656) = 49.21, *p* < .001, fitness evaluation: χ²[4] = 70.20, *p* < .001, health orientation: χ²[4] = 69.70, *p* < .001, illness orientation: *F*(4, 656) = 7.49, *p* < .001, and health evaluation: *F*(4, 217.18) = 33.20, *p* < .001). Post hoc tests revealed significant differences for most profile comparisons, with non-significant comparisons indicated by superscripts in Table 1 and full results provided in Table S7.

**Fig. 2:**
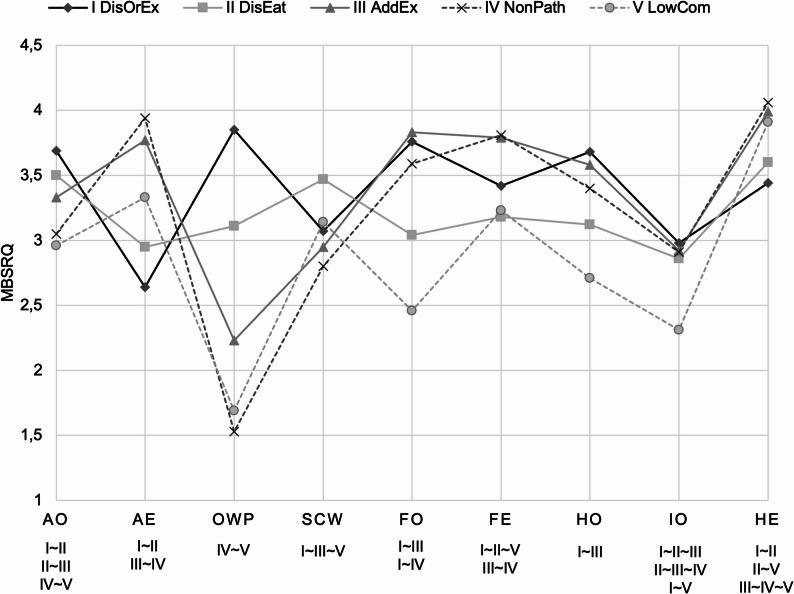
Mean values of the multidimensional body-self relations questionnaire scales across the five profiles. *Note*. AO = appearance orientation, AE = appearance evaluation, OWP = overweight preoccupation, SCW = self-classified weight, FO = fitness orientation, FE = fitness evaluation, HO = health orientation, IO = illness orientation, HE = health orientation. ~ = indicates non-significant differences between profiles based on post hoc tests; all other pairs differ significantly

Regarding self-esteem, post hoc tests revealed that participants in DisOrEx reported the significantly lowest RSES scores (*M* = 14.10; see Table 1). DisEat showed higher scores, but significantly lower than AddEx, LowCom, and NonPath in ascending order. The only nonsignificant RSES differences were between LowCom with AddEx (*M*_Δ_ = -0.68, 95%-CI [-3.48, 2.11]) and NonPath (*M*_Δ_ = -2.30, 95%-CI [-5.18, 0.58]). The profiles’ differing patterns across all MBSRQ scales are illustrated in Fig. 2, providing a visual overview of how multidimensional body image varies between profiles. For the appearance orientation scale, most profiles differed significantly, with the exceptions of DisOrEx vs. DisEat (*M*_Δ_ = -0.19, 95%-CI [-0.42, 0.33]), DisEat vs. AddEx (*M*_Δ_ = -0.16, 95%-CI [-0.35, 0.03]), and NonPath vs. LowCom (*M*_Δ_ = -0.09, 95%-CI [-0.38, 0.20]). Specifically, DisOrEx, DisEat, and AddEx exhibited significantly higher appearance orientation compared to NonPath and LowCom. Similarly, overweight preoccupation was highest in DisOrEx, followed by DisEat, AddEx, and NonPath / LowCom in descending order. Among these, only NonPath and LowCom did not differ significantly (*M*_Δ_ = 0.16, 95%-CI [-0.13, 0.46]). Among the MBSRQ scales, overweight preoccupation showed the largest variation across profiles, as depicted in Fig. 2. Thus, participants in all three profiles having above-average DOS, EDE-Q8, or EAI-R scores, although the degree of concern for weight differed among them, were more concerned with both appearance and weight than were people with profiles characterized by lower DOS, EDE-Q8, or EAI-R scores. In contrast, appearance evaluation was highest in AddEx and NonPath, intermediate in LowCom, and lowest in DisOrEx and DisEat. The only non-significant differences were between DisOrEx vs. DisEat (*M*_Δ_ = 0.31, 95%-CI [0.00, 0.62]) and AddEx vs. NonPath (*M*_Δ_ = 0.17, 95%-CI [0.00, 0.34]). Moreover, self-classified weight was highest in DisEat, followed by DisOrEx, AddEx and LowCom, which did not differ significantly from each other (DisOrEx vs. AddEx: *M*_Δ_ = -0.11, 95%-CI [-0.33, 0.10]; DisOrEx vs. LowCom: *M*_Δ_ = 0.07, 95%-CI [-0.24, 0.39]; AddEx vs. LowCom: *M*_Δ_ = 0.18, 95%-CI [-0.08, 0.45]), and lowest in NonPath.

Regarding fitness orientation, only DisOrEx did not significantly differ from AddEx (*M*_Δ_ = 0.07, 95%-CI [-0.17, 0.32]) and NonPath (*M*_Δ_ = -0.16, 95%-CI [-0.43, 0.10]). These three profiles exhibited the highest orientation towards fitness, followed by DisEat, and lastly LowCom. Likewise, both AddEx and NonPath (*M*_Δ_ = 0.02, 95%-CI [-0.18, 0.21]) reported significantly higher fitness evaluation than the other profiles, whereas DisOrEx had scores similar to those of DisEat and LowCom (DisOrEx vs. DisEat: *M*_Δ_ = -0.24, 95%-CI [-0.52, 0.05], DisOrEx vs. LowCom: *M*_Δ_ = -0.20, 95%-CI [-0.60, 0.21], DisEat vs. LowCom: *M*_Δ_ = 0.04, 95%-CI [-0.36, 0.44]). Health orientation was highest in DisOrEx and AddEx, which were the only profiles that did not significantly (*M*_Δ_ = -0.10, 95%-CI [-0.28, 0.09]), followed by NonPath, DisEat, and LowCom in decreasing order. In contrast, the profiles reported comparable levels of illness orientation, with LowCom being the only profile significantly less concerned than the others. Health evaluation was highest in profiles AddEx, NonPath, and LowCom, which did not significantly differ from each other (AddEx vs. NonPath: *M*_Δ_ = 0.07, 95%-CI [-0.12, 0.26], AddEx vs. LowCom: *M*_Δ_ = -0.08, 95%-CI [-0.38, 0.23], NonPath vs. LowCom: *M*_Δ_ = -0.15, 95%-CI [-0.47, 0.17]). Profiles DisOrEx and DisEat were less and similarily satisfied with their health (MΔ = 0.16, 95% CI [–0.14, 0.45]), while DisEat and LowCom did not differ significantly (*M*_Δ_ = 0.32, 95%-CI [-0.02, 0.65]). Across all health- and fitness-related body image scales, DisOrEx, AddEx, and NonPath shared more similarities with each other than with DisEat and LowCom, as illustrated in Fig. 2.

## Discussion

### Summary of main findings

This study examined whether Orthorexia Nervosa (ON) and Exercise Addiction (EA) can be distinguished from disordered eating by identifying latent profiles and comparing these profiles on multidimensional body image and self-esteem. The latent profile analysis yielded five distinct groups: (I) DisOrEx – Disordered and orthorexic eating behaviors accompanied by instrumental exercise (i.e., exercise performed primarily to control weight or shape), (II) DisEat – Disordered eating, (III) AddEx – Exercise Addiction, (IV) NonPath – Non-pathological eating behaviors, and (V) LowCom – Low commitment to both diet and exercise.

In our first hypothesis, we expected that profiles high in ON but low in disordered eating would show stronger health orientation. However, no such independent ON profile emerged, thus challenging the independence of ON from disordered eating. In this respect, we did not replicate the previously observed unique ON profile distinct from disordered eating [40] when accounting for measurement error and identifying latent subpopulations. Moreover, latent-level associations showed that both ON and EA were strongly linked to disordered eating, but had small overlap with each other. Taken together, these findings suggest that ON may be more closely aligned with disordered eating than previously assumed, rather than representing a clearly distinct eating disorder independent of weight and shape concerns [2]. Consistent with our second hypothesis – that EA-dominated profiles would be highly oriented to fitness – profile AddEx indeed exhibited high fitness orientation. AddEx was characterized by below-average disordered eating levels and modestly elevated EA levels. The prevalence of EA risk (6.5%) was low in comparison with 31.4% in profile DisOrEx. Moreover, EA levels were significantly higher in DisOrEx, suggesting that instrumental exercise appears to be associated with greater symptom severity than EA. This pattern aligns with a meta-analysis showing that EA is at least three times more likely to co-occur with disordered eating (i.e. instrumental exercise) than to occur in isolation [24]. Our third hypothesis – that profiles characterized by disordered eating alone would be most strongly associated with overweight and appearance concerns, lesser satisfaction with appearance, and lowest self-esteem – was not supported. While both DisOrEx and DisEat exhibited the highest appearance orientation and the lowest appearance evaluation, DisOrEx exceeded DisEat in overweight preoccupation and reduced self-esteem. Notably, the gender distribution differed across profiles: DisOrEx and DisEat were predominantly female, whereas AddEx, NonPath, and LowCom included a higher proportion of male participants. This pattern aligns with the generally higher prevalence of disordered eating and eating disorders in women, providing context for the observed profile distributions [14].

### Orthorexia nervosa and exercise addiction: a matter of appearance and overweight-preoccupation?

Although there is conceptual consensus that pronounced overvaluation of appearance and body concerns excludes ON diagnostically [5], we did not identify a latent subpopulation reflecting this pattern. Instead, participants in both profiles with elevated disordered eating levels, i.e. DisOrEx and DisEat, showed the highest preoccupation with their body’s appearance, while also reporting the lowest satisfaction with it. Notably, disordered eating severity was higher in profile DisOrEx than in DisEat. Consistent with this elevated symptom burden, 38.1% of participants in DisOrEx reported a current eating disorder diagnosis and 47.6% a past diagnosis, compared to 5.3% and 11.5% in profile DisEat, respectively. Additionally, overweight preoccupation was also highest in participants of DisOrEx, aligning with prior research showing that weight preoccupation predicts eating disorder onset [79–81] and relates to higher severity in patients with Anorexia Nervosa [82]. Given its previously reported association with overweight preoccupation [39, 40], ON may have contributed to the elevated levels in DisOrEx, beyond the impact of disordered eating. Despite these marked differences in symptom severity and overweight preoccupation, participants in DisOrEx and DisEat evaluated their appearance similarly. The low BMI in DisOrEx, with 16.2% of participants being underweight, may help account for these results. Since patients with eating disorders at low body weight do not necessarily experience greater appearance dissatisfaction than healthy controls, similar appearance evaluations are plausible [37].

In contrast, profile AddEx showed unique patterns in appearance-related body image. Participants in AddEx showed similar levels of appearance orientation as those in DisEat, but lower than DisOrEat. Overall, participants in AddEx had intermediate levels of overweight preoccupation – lower than both disordered eating profiles and higher than the “healthy” profiles. On the other side, they evaluated their appearance as highest, alongside NonPath. Thus, individuals at risk for EA prioritize appearance and weight but maintain satisfaction with their physical attractiveness as well, indicating a clear difference to instrumental exercise. Thus, the previously observed moderate correlations between EA and negative body image [33] may stem, at least in part, from the overlap of EA with instrumental exercise in eating disorders [11, 24]. With regard to self-esteem, participants displaying EA tendencies also differed substantially from those engaging in instrumental exercise.

### Self-esteem differences between participants showing instrumental exercise and exercise addiction

Although DisOrEx and AddEx profiles both showed elevated EA scores, only participants in DisOrEx reported decreased self-esteem levels, yielding the lowest values among all profiles. Specifically, the mean score in DisOrEx fell below the threshold for low self-esteem, with 52.4% of participants affected. In contrast, participants of AddEx exhibited the second highest self-esteem level, ranking just below the profile NonPath. These differences mirror the profiles’ appearance evaluation levels, consistent with evidence that greater self-esteem is related to higher body satisfaction [26]. Our findings suggest that, unlike instrumental exercise, EA is not accompanied by impaired self-esteem – further underscoring the importance of distinguishing between addictive and instrumental forms of exercise behavior [11]. Relatedly, Gori et al. [31] reported that the link between EA and body image concerns weakens as self-esteem increases. Since disordered eating was not accounted for in their study, these associations may in fact reflect instrumental exercise tied to disordered eating – rather than genuine EA. The EAI-R may lack specificity in that regard: 31.4% of participants in profile DisOrEx were classified as at risk for EA, whereas only 6.5% of those in profile AddEx received the same classification.

Moreover, the extent to which AddEx represents individuals at risk for EA warrants further consideration. Given that low self-esteem is a well-established transdiagnostic marker for psychopathology [83], it seems plausible that the modestly elevated EA scores in AddEx reflect a non-pathological form of exercise commitment or passion rather than clinically relevant EA [84, 85]. In contrast to EA, a passion for exercise reflects a strong yet flexible engagement: individuals dedicate substantial time and energy to physical activity, value it deeply, and derive enjoyment from it [86]. While such involvement can include elements of rigidity, pressure, or intense striving – particularly among intensely passionate exercisers – it does not necessarily imply loss of control or psychological distress [87]. This interpretation aligns with findings in female athletes who, despite being classified as at risk for EA, showed no signs of withdrawal or psychological distress when disordered eating was absent [88]. However, our study does not permit conclusions regarding the presence or extent of functional impairments or distress related to exercise behavior in the participants. Furthermore, unlike participants engaging in instrumental exercise, those in the AddEx profile reported high satisfaction with their health and fitness, as discussed in the following section.

### The roles of fitness, health and illness in body image

Interestingly, profiles with elevated ON or EA levels exhibited the greatest cognitive and behavioral investment in health and fitness – even in the presence of disordered eating. While participants in profiles DisOrEx, AddEx and NonPath placed a high importance on feeling physically fit or athletic, participants with symptoms of disordered eating only did not. Notably, participants in AddEx showed only slightly higher fitness orientation than NonPath, suggesting that EA may not mainly arise from an (exaggerated) desire to feel fit or athletic, but rather from mood and affect regulation, for example [89, 90]. Fitness evaluation, rather than fitness orientation, distinguished participants with EA tendencies from those engaging in instrumental exercise. Although both groups invested in fitness, only individuals with EA were satisfied with it. A similar pattern emerged for health orientation, which differed among participants with disordered eating depending on the presence of ON symptoms and instrumental exercise. Participants in both DisOrEx and DisEat rated their satisfaction with health as low, whereas only those in DisOrEx placed emphasis on it. In contrast, both health orientation and evaluation were high in AddEx. Conversely, illness orientation failed to differentiate between profiles except for LowCom, contradicting prior research reporting small to moderate associations between ON and illness anxiety [91–93]. Hence, orthorexic tendencies may stem from a strong belief in in the individual controllability of health rather than heightened alertness or reactivity to bodily symptoms [94].

### Limitations

This study provides novel insights in multidimensional body image in ON and EA, overcoming previous limitations of using invalid ON measures [51, 95]. To our knowledge, this is the first study examining body image in EA beyond appearance, highlighting the need for further research in this area. The latent profile analysis with a large sample size allowed for nuanced profile differentiation, addressing measurement error and revealing underlying patterns. However, the profile solution was not cross-validated, and the results may be sensitive to the specific characteristics of our sample, which included a relatively high proportion of female participants. Consequently, the stability and generalizability of the identified profiles to other populations, particularly more gender-balanced or -specific samples, remains uncertain. We note that issues of the factor structure of the questionnaires DOS, EAI-R, and EDE-Q8 warrant further examination. Overall, the findings suggest that the EAI-R may lack specificity, often capturing instrumental exercise rather than symptoms of a behavioral addiction. Moreover, the high uniqueness and residual variances of most DOS items suggest potential confounding, such as a non-pathological interest in healthy eating [21, 25]. Notably, the item measuring a positive attitude towards healthy eating was the least well-represented by the factor structure, further supporting this interpretation. Another limitation is that the use of convenience sampling, combined with the study title, likely resulted in a predominantly female, highly educated sample, with notable rates of participants reporting current eating disorders (7.9%), and risks for ON (8.5%) or EA (7.5%). In addition to this selection bias, social desirability may have biased responses on the explicit self-report measures. Furthermore, the cross-sectional design does not allow any causal conclusions regarding the nature of relationships of ON, EA, disordered eating with body image and self-esteem, which may serve as risk, outcome, or maintenance factors [96, 97]. Overall, these findings emphasize the importance of assessing disordered eating when examining ON and EA.

## Conclusion

The present findings challenge the notion that ON is independent from disordered eating, supporting previous concerns on this issue [2]. Instead, ON could represent a more socially acceptable manifestation of disordered eating, in which individuals disguise unhealthy behaviors as health-conscious choices rather than appearance-driven actions [22, 23, 98]. Although EA formed a distinct profile characterized by positive body image, its modestly elevated EA levels and high self-esteem highlight the need to clarify whether EA reflects a clinically relevant construct or simply a strong passion for exercise [85]. Notably, participants with the most pronounced symptoms of disordered eating, ON, and instrumental exercise exhibited the poorest body image and self-esteem. Both EA and ON may exacerbate negative body image in individuals with disordered eating by adding pressures not only to appearance, but also to the maintenance of physical fitness and overall health. Because disordered eating can impair physical fitness and health while simultaneously provoking weight anxiety [99–101], intraindividual conflicts may arise. Shifting beauty ideals may also contribute, as women increasingly prefer athletic bodies over mere thinness [102]. Supporting this trend, “fitspiration” content on social media, intended to encourage fitness and health, predominantly emphasize appearance [103]. Creators of such content, regardless of gender, tend to display stronger drives for thinness and muscularity, along with higher tendencies toward disordered eating and instrumental exercise [104]. Consequently, the aspiration to look fit and healthy – rather than actually achieving it – may shape disordered eating and negative body image in modern Western societies. In treatment interventions, the pronounced orientation toward health and fitness could be leveraged as a positive resource, redirecting patients’ efforts toward adaptive goals instead of maladaptive behaviors focused on weight and appearance.

## Electronic Supplementary Material

Below is the link to the electronic supplementary material.

Supplementary Material 1

## Data Availability

The datasets analyzed during the current study are available from the corresponding author on request.

## Abbreviations

AddEx: Exercise Addiction Profile
AIC: Akaike’s Information Criterion
AN: Anorexia Nervosa
AWE: Approximate Weight of Evidence
BIC: Bayesian Information Criterion
BN: Bulimia Nervosa
BSA-F: Physical Activity, Exercise, and Sport Questionnaire (Bewegungs- und Sportaktivität Fragebogen)
CFI: Comparative Fit Index
CLC: Classification Likelihood Criterion
DisEat: Disordered Eating Profile
DisOrEx: Disordered and Orthorexic Eating Behaviors with Instrumental Exercise Profile
DOS: Düsseldorf Orthorexia Scale
EA: Exercise Addiction
EAI-R: Revised Exercise Addiction Inventory
EDE-Q8: Short Eating Disorder Examination Questionnaire
KIC: Kullback Information Criterion
LowCom: Low Commitment to Diet and Exercise Profile
MBSRQ: Multidimensional Body-Self Relations Questionnaire
NonPath: Non-Pathological Eating Behaviors Profile
ON: Orthorexia Nervosa
RMSEA: Root Mean Square Error of Approximation
RSES: Rosenberg Self-Esteem Scale
SRMR: Standardized Root Mean Square Residual

## References

[1] Godoy-Izquierdo D, Ramírez MJ, Díaz I, López-Mora C. A Systematic Review on Exercise Addiction and the Disordered Eating-Eating Disorders Continuum in the Competitive Sport Context. Int J Ment Health Addict. 2023;21(1):529–61. 10.1007/s11469-021-00610-2.

[2] Meule A, Voderholzer U. Orthorexia Nervosa - It Is Time to Think About Abandoning the Concept of a Distinct Diagnosis. Front Psychiatry. 2021;12:640401. 10.3389/fpsyt.2021.640401. PubMed PMID: 33584391. 33584391 10.3389/fpsyt.2021.640401 PMC7876234

[3] American Psychiatric Association, editor. Diagnostic and statistical manual of mental disorders (DSM-5^®^). American Psychiatric Pub. 2013.

[4] World Health Organization. World Health Organization [Internet]. 2019. International classification of diseases for mortality and morbidity statistics (11th Revision). Available from: https://icd.who.int/.

[5] Donini LM, Barrada JR, Barthels F, Dunn TM, Babeau C, Brytek-Matera A, et al. A consensus document on definition and diagnostic criteria for orthorexia nervosa. Eat Weight Disord EWD. 2022;27(8):3695–711. 10.1007/s40519-022-01512-5. PubMed PMID: 36436144. 36436144 10.1007/s40519-022-01512-5 PMC9803763

[6] Cena H, Barthels F, Cuzzolaro M, Bratman S, Brytek-Matera A, Dunn T, et al. Definition and diagnostic criteria for orthorexia nervosa: a narrative review of the literature. Eat Eeight Disord EWD. 2019;24(2):209–46. 10.1007/s40519-018-0606-y. PubMed PMID: 30414078. 30414078 10.1007/s40519-018-0606-y

[7] Griffiths M. Exercise Addiction: A Case Study. Addict Res. 1997;5(2):161–8. 10.3109/16066359709005257.

[8] Hausenblas HA, Downs DS. How Much is Too Much? The Development and Validation of the Exercise Dependence Scale. Psychol Health. 2002;17(4):387–404. 10.1080/0887044022000004894.

[9] Niedzielski A, Kaźmierczak-Wojtaś N. Prevalence of orthorexia nervosa and its diagnostic tools-a literature review. Int J Environ Res Public Health. 2021;18(10). 10.3390/ijerph18105488. PubMed PMID: 34065506. 10.3390/ijerph18105488 PMC8160773 34065506

[10] Strahler J. Sex differences in orthorexic eating behaviors: A systematic review and meta-analytical integration. Nutr Burbank Los Angel Cty Calif. 2019;67–68:110534. 10.1016/j.nut.2019.06.015 PubMed PMID: 31525607. 10.1016/j.nut.2019.06.015 31525607

[11] Weinstein A, Szabo A. Exercise addiction: A narrative overview of research issues. Dialogues Clin Neurosci. 2023;25(1):1–13. 10.1080/19585969.2023.2164841. PubMed PMID: 36698618. 36698618 10.1080/19585969.2023.2164841 PMC9869993

[12] Di Lodovico L, Poulnais S, Gorwood P. Which sports are more at risk of physical exercise addiction: A systematic review. Addict Behav. 2019;93:257–62. 10.1016/j.addbeh.2018.12.030. 30595420 10.1016/j.addbeh.2018.12.030

[13] Müller A, Cook B, Zander H, Herberg A, Müller V, De Zwaan M. Does the German version of the Exercise Dependence Scale measure exercise dependence? Psychol Sport Exerc. 2014;15(3):288–92. 10.1016/j.psychsport.2013.12.003.

[14] Galmiche M, Déchelotte P, Lambert G, Tavolacci MP. Prevalence of eating disorders over the 2000–2018 period: a systematic literature review. Am J Clin Nutr. 2019;109(5):1402–13. 10.1093/ajcn/nqy342. 31051507 10.1093/ajcn/nqy342

[15] Richter F, Strauss B, Braehler E, Adametz L, Berger U. Screening disordered eating in a representative sample of the German population: Usefulness and psychometric properties of the German SCOFF questionnaire. Eat Behav. 2017;25:81–8. 10.1016/j.eatbeh.2016.06.022. PubMed PMID: 27354266. 27354266 10.1016/j.eatbeh.2016.06.022

[16] Strahler J, Wachten H, Mueller-Alcazar A. Obsessive healthy eating and orthorexic eating tendencies in sport and exercise contexts: a systematic review and meta-analysis. J Behav Addict. 2021 Jan;1. 10.1556/2006.2021.00004. PubMed PMID: 33650987. 10.1556/2006.2021.00004 PMC8997206 33650987

[17] Cheshire A, Berry M, Fixsen A. What are the key features of orthorexia nervosa and influences on its development? A qualitative investigation. Appetite. 2020;155:104798. 10.1016/j.appet.2020.104798. PubMed PMID: 32717291. 32717291 10.1016/j.appet.2020.104798

[18] Miller ML, Hormes JM. Cognitive and Behavioral Inflexibility as a Transdiagnostic Process Underpinning Exercise Dependence. Int J Ment Health Addict. 2023;21(5):3446–57. 10.1007/s11469-022-00802-4.

[19] Campbell C, Greig X, Griffiths J, Hashman D, Sottile T, Isobe M, et al. The prevalence of excessive exercise in eating disorders: a systematic review and meta-analysis. 33. 2026;33(1005–16). 10.17605/OSF.IO/MYVXW. 10.1002/erv.3194 PMC12319126 40252210

[20] Atchison AE, Zickgraf HF. Orthorexia nervosa and eating disorder behaviors: A systematic review of the literature. Appetite. 2022;177:106134. 10.1016/j.appet.2022.106134. PubMed PMID: 35750289. 35750289 10.1016/j.appet.2022.106134

[21] Zickgraf HF, Ellis JM, Essayli JH. Disentangling orthorexia nervosa from healthy eating and other eating disorder symptoms: Relationships with clinical impairment, comorbidity, and self-reported food choices. Appetite. 2019;134:40–9. 10.1016/j.appet. 2018.12.006 PubMed PMID: 30543837. 30543837 10.1016/j.appet.2018.12.006 PMC8056745

[22] Bartel SJ, Sherry SB, Farthing GR, Stewart SH. Classification of Orthorexia Nervosa: Further evidence for placement within the eating disorders spectrum. Eat Behav. 2020;38:101406. 10.1016/j.eatbeh.2020.101406. PubMed PMID: 32540715. 32540715 10.1016/j.eatbeh.2020.101406

[23] Depa J, Barrada JR, Roncero M. Are the motives for food choices different in orthorexia nervosa and healthy orthorexia? Nutrients. 2019;11(3). 10.3390/nu11030697. PubMed PMID: 30934544. 10.3390/nu11030697 PMC6470526 30934544

[24] Trott M, Jackson SE, Firth J, Jacob L, Grabovac I, Mistry A, et al. A comparative meta-analysis of the prevalence of exercise addiction in adults with and without indicated eating disorders. Eat Weight Disord EWD. 2021;26(1):37–46. 10.1007/s40519-019-00842-1. PubMed PMID: 31894540. 31894540 10.1007/s40519-019-00842-1

[25] Zagaria A, Vacca M, Cerolini S, Ballesio A, Lombardo C. Associations between orthorexia, disordered eating, and obsessive-compulsive symptoms: A systematic review and meta-analysis. Int J Eat Disord. 2022;55(3):295–312. 10.1002/eat.23654. PubMed PMID: 34921564. 34921564 10.1002/eat.23654

[26] Mellor D, Fuller-Tyszkiewicz M, McCabe MP, Ricciardelli LA. Body Image and Self-Esteem Across Age and Gender: A Short-Term Longitudinal Study. Sex Roles. 2010;63(9–10):672–81. 10.1007/s11199-010-9813-3.

[27] Vartanian LR, Smyth JM, Zawadzki MJ, Heron KE, Coleman SRM. Early adversity, personal resources, body dissatisfaction, and disordered eating. Int J Eat Disord. 2014;47(6):620–9. 10.1002/eat.22313. PubMed PMID: 24902671. 24902671 10.1002/eat.22313 PMC10685386

[28] Bóna E, Erdész A, Túry F. Low self-esteem predicts orthorexia nervosa, mediated by spiritual attitudes among frequent exercisers. Eat Weight Disord EWD. 2021;26(8):2481–9. 10.1007/s40519-020-01095-z. PubMed PMID: 33502732. 33502732 10.1007/s40519-020-01095-z PMC8602160

[29] Yakın E, Raynal P, Chabrol H. Distinguishing between healthy and pathological orthorexia: a cluster analytic study. Eat Weight Disord EWD. 2022;27(1):325–34. 10.1007/s40519-021-01178-5. PubMed PMID: 33826119. 33826119 10.1007/s40519-021-01178-5

[30] Bruno A, Quattrone D, Scimeca G, Cicciarelli C, Romeo VM, Pandolfo G, et al. Unraveling exercise addiction: the role of narcissism and self-esteem. J Addict. 2014;2014:987841. 10.1155/2014/987841. PubMed PMID: 25405056. 25405056 10.1155/2014/987841 PMC4227365

[31] Gori A, Topino E, Griffiths MD. Protective and risk factors in exercise addiction: a series of moderated mediation analyses. Int J Environ Res Public Health. 2021;18(18). 10.3390/ijerph18189706. PubMed PMID: 34574631. 10.3390/ijerph18189706 PMC8467293 34574631

[32] Alcaraz-Ibáñez M, Paterna A, Sicilia Á, Griffiths MD. A Systematic Review and Meta-Analysis on the Relationship between Body Dissatisfaction and Morbid Exercise Behaviour. Int J Environ Res Public Health. 2021;18(2):585. 10.3390/ijerph18020585. 33445591 10.3390/ijerph18020585 PMC7827926

[33] Guo S, Kamionka A, Xue Q, Izydorczyk B, Lipowska M, Lipowski M. Body image and risk of exercise addiction in adults: a systematic review and meta-analysis. J Behav Addict. 2025 Feb;6. 10.1556/2006.2024.00085 10.1556/2006.2024.00085 PMC11974424 39912824

[34] Ahorsu DK, Imani V, Potenza MN, Chen HP, Lin CY, Pakpour AH. Mediating Roles of Psychological Distress, Insomnia, and Body Image Concerns in the Association Between Exercise Addiction and Eating Disorders. Psychol Res Behav Manag. 2023;16:2533–42. 10.2147/PRBM.S414543%23d1e264 PubMed PMID: 37431433. 37431433 10.2147/PRBM.S414543 PMC10329837

[35] Cash TF. Body image: past, present, and future. Body Image. 2004;1(1):1–5. 10.1016/S1740-1445(03)00011-1. 18089136 10.1016/S1740-1445(03)00011-1

[36] Cash TF. Multidimensional Body–Self Relations Questionnaire (MBSRQ). In: Wade T, editor. Encyclopedia of feeding and eating disorders. Singapore: Springer Singapore; 2016. pp. 1–4. 10.1007/978-981-287-087-2_3-1.

[37] Prnjak K, Jukic I, Mitchison D, Griffiths S, Hay P. Body image as a multidimensional concept: A systematic review of body image facets in eating disorders and muscle dysmorphia. Body Image. 2022;42:347–60. 10.1016/j.bodyim.2022.07.006. PubMed PMID: 35926364. 35926364 10.1016/j.bodyim.2022.07.006

[38] Hrabosky JI, Cash TF, Veale D, Neziroglu F, Soll EA, Garner DM, et al. Multidimensional body image comparisons among patients with eating disorders, body dysmorphic disorder, and clinical controls: a multisite study. Body Image. 2009;6(3):155–63. 10.1016/j.bodyim.2009.03.001. PubMed PMID: 19410528. 19410528 10.1016/j.bodyim.2009.03.001

[39] Pauzé A, Plouffe-Demers MP, Fiset D, Saint-Amour D, Cyr C, Blais C. The relationship between orthorexia nervosa symptomatology and body image attitudes and distortion. Sci Rep. 2021;11(1):13311. 10.1038/s41598-021-92569-2. PubMed PMID: 34172763. 34172763 10.1038/s41598-021-92569-2 PMC8233361

[40] Yakın E, Raynal P, Chabrol H. Distinguishing orthorexic behaviors from eating disordered and obsessive-compulsive behaviors: a typological study. Eat Weight Disord EWD. 2021a;26(6):2011–9. 10.1007/s40519-020-01037-9. PubMed PMID: 33111166. 33111166 10.1007/s40519-020-01037-9

[41] Aiello P, Toti E, Villaño D, Raguzzini A, Peluso I. Overlap of orthorexia, eating attitude and psychological distress in some Italian and Spanish university students. World J Psychiatry. 2022;12(10):1298–312. 10.5498/wjp.v12.i10.1298. 36389086 10.5498/wjp.v12.i10.1298 PMC9641377

[42] Barnes MA, Caltabiano ML. The interrelationship between orthorexia nervosa, perfectionism, body image and attachment style. Eat Weight Disord EWD. 2017;22(1):177–84. 10.1007/s40519-016-0280. -x PubMed PMID: 27068175. 27068175 10.1007/s40519-016-0280-x

[43] Brytek-Matera A, Donini LM, Krupa M, Poggiogalle E, Hay P. Orthorexia nervosa and self-attitudinal aspects of body image in female and male university students. J Eat Disord. 2015;3:2. 10.1186/s40337-015-0038-2. PubMed PMID: 25774296. 25774296 10.1186/s40337-015-0038-2 PMC4359442

[44] Brytek-Matera A, Rogoza R, Gramaglia C, Zeppegno P. Predictors of orthorexic behaviours in patients with eating disorders: a preliminary study. BMC Psychiatry. 2015;15:252. 10.1186/s12888-015-0628-1. PubMed PMID: 26472110. 26472110 10.1186/s12888-015-0628-1 PMC4608153

[45] Mitrofanova E, Mulrooney H, Petróczi A. Assessing psychological and nutritional impact of suspected orthorexia nervosa: a cross-sectional pilot study. J Hum Nutr Diet Off J Br Diet Assoc. 2021;34(1):42–53. 10.1111/jhn.12797. PubMed PMID: 33216395. 10.1111/jhn.12797 33216395

[46] Leiner DJ. Too fast, too straight, too weird: non-reactive indicators for meaningless data in internet surveys. Surv Res Methods. 2019;229–48. 10.18148/SRM/2019.V13I3.7403.

[47] Wachten H, Wurst R, Paganini S, Strahler J. Excessive health behaviors in sports: links of orthorexia nervosa and exercise addiction with well-being, exercise activity in sports categories, and gender effects. Front Nutr. 2024;11:1494958. 10.3389/fnut.2024.1494958. 39691172 10.3389/fnut.2024.1494958 PMC11649418

[48] Caspersen CJ, Powell KE, Christenson GM. Physical activity, exercise, and physical fitness: definitions and distinctions for health-related research. Public Health Rep Wash DC 1974. 1985;100(2):126–31. PubMed PMID: 3920711; PubMed Central PMCID: PMC1424733. PMC1424733 3920711

[49] Sehlbrede M, Wurst R. fitevalapp [Internet]. 2022. Available from: Available from: https://github.com/Methodicum/fitevalapp

[50] Barthels F, Meyer F, Pietrowsky R. Die Düsseldorfer Orthorexie Skala–Konstruktion und Evaluation eines Fragebogens zur Erfassung ortho-rektischen Ernährungsverhaltens. Zeitschrift für Klinische Psychologie und Psychotherapie. 2015;97–105.

[51] Opitz MC, Newman E, Alvarado Vázquez Mellado AS, Robertson MDA, Sharpe H. The psychometric properties of Orthorexia Nervosa assessment scales: A systematic review and reliability generalization. Appetite. 2020;155:104797. 10.1016/j.appet.2020.104797. PubMed PMID: 32652100. 32652100 10.1016/j.appet.2020.104797

[52] Valente M, Syurina EV, Donini LM. Shedding light upon various tools to assess orthorexia nervosa: a critical literature review with a systematic search. Eat Weight Disord EWD. 2019;24(4):671–82. 10.1007/s40519-019-00735-3. PubMed PMID: 31228168. 31228168 10.1007/s40519-019-00735-3 PMC6647444

[53] Szabo A, Pinto A, Griffiths MD, Kovácsik R, Demetrovics Z. The psychometric evaluation of the Revised Exercise Addiction Inventory: Improved psychometric properties by changing item response rating. J Behav Addict. 2019;8(1):157–61. 30920295 10.1556/2006.8.2019.06 PMC7044604

[54] Griffiths M. A ‘components’ model of addiction within a biopsychosocial framework. J Subst Use. 2005;10(4):191–7. 10.1080/14659890500114359.

[55] Terry A, Szabo A, Griffiths M. The exercise addiction inventory: a new brief screening tool. Addict Res Theory. 2004;12(5):489–99. 10.1080/16066350310001637363.

[56] Kliem S, Mößle T, Zenger M, Strauß B, Brähler E, Hilbert A. The eating disorder examination-questionnaire 8: A brief measure of eating disorder psychopathology (EDE-Q8). Int J Eat Disord. 2016;49(6):613–6. 10.1002/eat.22487. PubMed PMID: 26711183. 26711183 10.1002/eat.22487

[57] Hilbert A, Tuschen-Caffier B. Eating Disorder Examination. 2. Auflage. Tübingen: dgvt-Verlag; 2016.

[58] Cash TF. Multidimensional body-self relations questionnaire: MBSRQ user’s manual. Norfolk VA Old Dom Univ. 2000 Jan 1.

[59] Mühlan H, Schmidt SMBSRQ. Multidimensional Body Self Relations Questionnaire - Deutsche Version. In: Kupfer J, Schmidt S, Augustin M, editors. Psychodiagnostische Verfahren für die Dermatologie. Göttingen: Hogrefe; 2006. (Diagnostik für Klinik und Praxis).

[60] Rosenberg M. Rosenberg self-esteem scale (RSE). Accept Commit Ther Meas Package. 1965;61(52):18.

[61] Collani G, Herzberg PY. Eine revidierte Fassung der deutschsprachigen Skala zum Selbstwertgefühl von Rosenberg. Z Für Differ Diagn Psychol. 2003;24(1):3–7.

[62] R Core Team. R: A Language and environment for statistical computing [Internet]. Vienna, Austria. 2022. Available from: https://www.R-project.org/.

[63] Zhang Y, Zhou M, Shao Y, mvnormalTest. powerful tests for multivariate normality [Internet]. 2020. Available from: https://CRAN.R-project.org/packagemvnormalTest.

[64] Zhou M, Shao Y. A Powerful Test for Multivariate Normality. J Appl Stat. 2014;41(2):351–63.839637 PubMed PMID: 24563571. 24563571 10.1080/02664763.2013.839637 PMC3927875

[65] Li CH. Confirmatory factor analysis with ordinal data: Comparing robust maximum likelihood and diagonally weighted least squares. Behav Res Methods. 2016;48(3):936–49. 10.3758/s13428-015-0619-7. PubMed PMID: 26174714. 26174714 10.3758/s13428-015-0619-7

[66] Rhemtulla M, Brosseau-Liard PÉ, Savalei V. When can categorical variables be treated as continuous? A comparison of robust continuous and categorical SEM estimation methods under suboptimal conditions. Psychol Methods. 2012;17(3):354–73. 10.1037/a0029315. PubMed PMID: 22799625. 22799625 10.1037/a0029315

[67] Hooper D, Coughlan J, Mullen M. Structural Equation modeling: guidelines for determining model fit. Electron J Bus Res Methods. 2008;6.

[68] Spurk D, Hirschi A, Wang M, Valero D, Kauffeld S. Latent profile analysis: A review and how to guide of its application within vocational behavior research. J Vocat Behav. 2020;120:103445. 10.1016/j.jvb.2020.103445.

[69] Rosenberg J, Beymer P, Anderson D, van Lissa C j., Schmidt J, tidyLPA. An R Package to easily carry out latent profile analysis (lpa) using open-source or commercial software. J Open Source Softw. 2018;3(30):978. 10.21105/joss.00978

[70] Nylund KL, Asparouhov T, Muthén BO. Deciding on the Number of Classes in Latent Class Analysis and Growth Mixture Modeling: A Monte Carlo Simulation Study. Struct Equ Model Multidiscip J. 2007;14(4):535–69. 10.1080/10705510701575396.

[71] McLachlan GJ, Peel D. Finite mixture models. Wiley. 2000.

[72] Akogul S, Erisoglu M. An Approach for Determining the Number of Clusters in a Model-Based Cluster Analysis. Entropy. 2017;19(9):452. 10.3390/e19090452.

[73] Blanca M, Alarcón R, Arnau J, Bono R, Bendayan R. Non-normal data: Is ANOVA still a valid option? Psicothema. 2017;4(29):552–7. 10.7334/psicothema2016.383. 10.7334/psicothema2016.383 29048317

[74] Lix LM, Keselman JC, Keselman HJ. Consequences of Assumption Violations Revisited: A Quantitative Review of Alternatives to the One-Way Analysis of Variance *F* Test. Rev Educ Res. 1996;66(4):579–619. 10.3102/00346543066004579.

[75] Games PA, Keselman HJ, Clinch JJ. Tests for homogeneity of variance in factorial designs. Psychol Bull. 1979;86(5):978–84. 10.1037/0033-2909.86.5.978.

[76] Guadagnoli E, Velicer WF. Relation of sample size to the stability of component patterns. Psychol Bull. 1988;103(2):265–75. 10.1037/0033-2909.103.2.265. PubMed PMID: 3363047. 3363047 10.1037/0033-2909.103.2.265

[77] Stevens JP. Applied Multivariate Statistics for the Social Sciences. Psychology; 2001. 10.4324/9781410604491.

[78] Jung T, Wickrama KAS. An Introduction to Latent Class Growth Analysis and Growth Mixture Modeling. Soc Personal Psychol Compass. 2008;2(1):302–17. 10.1111/j.1751-9004.2007.00054.x.

[79] Askew AJ, Peterson CB, Crow SJ, Mitchell JE, Halmi KA, Agras WS, et al. Not all body image constructs are created equal: Predicting eating disorder outcomes from preoccupation, dissatisfaction, and overvaluation. Int J Eat Disord. 2020;53(6):954–63. 10.1002/eat.23277. 32304257 10.1002/eat.23277 PMC9382219

[80] Mitchison D, Rieger E, Harrison C, Murray SB, Griffiths S, Mond J. Indicators of clinical significance among women in the community with binge-eating disorder symptoms: Delineating the roles of binge frequency, body mass index, and overvaluation. Int J Eat Disord. 2018;51(2):165–9. 10.1002/eat.22812. 29278426 10.1002/eat.22812

[81] Sharpe H, Griffiths S, Choo T, Eisenberg ME, Mitchison D, Wall M, et al. The relative importance of dissatisfaction, overvaluation and preoccupation with weight and shape for predicting onset of disordered eating behaviors and depressive symptoms over 15 years. Int J Eat Disord. 2018;51(10):1168–75. 10.1002/eat.22936. 30194690 10.1002/eat.22936 PMC6289784

[82] Linardon J, Phillipou A, Castle D, Newton R, Harrison P, Cistullo LL, et al. The relative associations of shape and weight over-evaluation, preoccupation, dissatisfaction, and fear of weight gain with measures of psychopathology: An extension study in individuals with anorexia nervosa. Eat Behav. 2018;29:54–8. 10.1016/j.eatbeh.2018.03.002. 29518651 10.1016/j.eatbeh.2018.03.002

[83] Zeigler-Hill V. The Connections Between Self-Esteem and Psychopathology. J Contemp Psychother. 2011;41(3):157–64. 10.1007/s10879-010-9167-8.

[84] La Vega R, Parastatidou IS, Roberto Ruíz-Barquín, Szabo A. Exercise Addiction in Athletes and Leisure Exercisers: The Moderating Role of Passion. J Behav Addict. 2016;5(2):325–31. 10.1556/2006.5.2016.043. PubMed PMID: 27363466. 27363466 10.1556/2006.5.2016.043 PMC5387784

[85] Szabo A, Kovacsik R. When Passion Appears, Exercise Addiction Disappears: Should Hundreds of Studies Not Considering Passion Be Revisited? Swiss J Psychol. 2019;78(3–4):137–42. 10.1024/1421-0185/a000228.

[86] Vallerand RJ, Blanchard C, Mageau GA, Koestner R, Ratelle C, Léonard M, et al. Les passions de l’âme: On obsessive and harmonious passion. J Pers Soc Psychol. 2003;85(4):756–67. 10.1037/0022-3514.85.4.756. 14561128 10.1037/0022-3514.85.4.756

[87] Szabo A, De La Vega R, Kovácsik R, Jiménez Almendros L, Ruíz-Barquín R, Demetrovics Z, et al. Dimensions of passion and their relationship to the risk of exercise addiction: Cultural and gender differences. Addict Behav Rep. 2022;16:100451. 10.1016/j.abrep.2022.100451. 36092546 10.1016/j.abrep.2022.100451 PMC9450070

[88] Bamber D, Im Cockerill, Rodgers S, Carroll D. It’s exercise or nothing: a qualitative analysis of exercise dependence. Br J Sports Med. 2000;34(6):423–30. 11131229 10.1136/bjsm.34.6.423 PMC1724254

[89] Bratland-Sanda S, Martinsen EW, Rosenvinge JH, Rø Ø, Hoffart A, Sundgot‐Borgen J. Exercise dependence score in patients with longstanding eating disorders and controls: The importance of affect regulation and physical activity intensity. Eur Eat Disord Rev. 2011;19(3):249–55. 10.1002/erv.971. 21584917 10.1002/erv.971

[90] Trott M, Yang L, Jackson SE, Firth J, Gillvray C, Stubbs B, et al. Prevalence and Correlates of Exercise Addiction in the Presence vs. Absence of Indicated Eating Disorders. Front Sports Act Living. 2020;2:84. 10.3389/fspor.2020.00084. PubMed PMID: 33345075. 33345075 10.3389/fspor.2020.00084 PMC7739814

[91] Barlow IU, Lee E, Saling L. Orthorexia nervosa versus healthy orthorexia: Anxiety, perfectionism, and mindfulness as risk and preventative factors of distress. Eur Eat Disord Rev. 2024;32(1):130–47. 10.1002/erv.3032. 37670425 10.1002/erv.3032

[92] Barthels F, Horn S, Pietrowsky R. Orthorexic eating behaviour, illness anxiety and dysfunctional cognitions characteristic of somatic symptom disorders in a non-clinical sample. Eat Weight Disord EWD. 2021 Jan;1. 10.1007/s40519-020-01091-3. PubMed PMID: 33392953. 10.1007/s40519-020-01091-3 33392953

[93] Chace S, Kluck AS. Validation of the Teruel Orthorexia Scale and relationship to health anxiety in a U.S. sample. Eat Weight Disord - Stud Anorex Bulim Obes. 2022;27(4):1437–47. 10.1007/s40519-021-01272-8. 10.1007/s40519-021-01272-8 34379313

[94] Greville-harris M, Talbot CV, Moseley RL, Vuillier L. Conceptualisations of health in orthorexia nervosa: a mixed-methods study. Eat Weight Disord - Stud Anorex Bulim Obes. 2022;27(8):3135–43. 10.1007/s40519-022-01443-1. 10.1007/s40519-022-01443-1 PMC9301897 35861935

[95] Barrada JR, Meule A. Orthorexia nervosa: research based on invalid measures is invalid. PsyArXiv; 2023. 10.31234/osf.io/39mwz. 10.7189/jogh.14.03007 PMC10835515 38304978

[96] Krauss S, Dapp LC, Orth U. The Link Between Low Self-Esteem and Eating Disorders: A Meta-Analysis of Longitudinal Studies. Clin Psychol Sci. 2023;11(6):1141–58. 10.1177/21677026221144255.

[97] Stice E, Interactive, Mediational Etiologic Models of Eating Disorder Onset. Evidence from Prospective Studies. Annu Rev Clin Psychol. 2016;12:359–81. 10.1146/annurev-clinpsy-021815-093317. PubMed PMID: 26651521. 26651521 10.1146/annurev-clinpsy-021815-093317

[98] Segura-Garcia C, Ramacciotti C, Rania M, Aloi M, Caroleo M, Bruni A, et al. The prevalence of orthorexia nervosa among eating disorder patients after treatment. Eat Weight Disord-Stud Anorex Bulim Obes. 2015;20(2):161–6. 10.1007/s40519-014-0171-y 25543324

[99] El Ghoch M, Soave F, Calugi S, Dalle Grave R. Eating disorders, physical fitness and sport performance: a systematic review. Nutrients. 2013;5(12):5140–60. doi:10.3390/nu5125140 PubMed PMID: 24352092. 24352092 10.3390/nu5125140 PMC3875919

[100] Kärkkäinen U, Mustelin L, Raevuori A, Kaprio J, Keski-Rahkonen A. Do Disordered Eating Behaviours Have Long‐term Health‐related Consequences? Eur Eat Disord Rev. 2018;26(1):22–8. 10.1002/erv.2568. 29160017 10.1002/erv.2568 PMC5732059

[101] López-Gil JF, García-Hermoso A, Smith L, Trott M, López-Bueno R, Gutiérrez-Espinoza H, et al. Physical fitness and disordered eating among adolescents: Results from the EHDLA study. Appetite. 2022;178:106272. 10.1016/j.appet.2022.106272. 35964793 10.1016/j.appet.2022.106272

[102] Bozsik F, Whisenhunt BL, Hudson DL, Bennett B, Lundgren JD. Thin Is In? Think Again: The Rising Importance of Muscularity in the Thin Ideal Female Body. Sex Roles. 2018;79(9–10):609–15. 10.1007/s11199-017-0886-0.

[103] Simpson CC, Mazzeo SE. Attitudes toward orthorexia nervosa relative to DSM-5 eating disorders. Int J Eat Disord. 2017;50(7):781–92. 10.1002/eat.22710. PubMed PMID: 28370208. 28370208 10.1002/eat.22710

[104] Holland G, Tiggemann M. A systematic review of the impact of the use of social networking sites on body image and disordered eating outcomes. Body Image. 2016;17:100–10. 10.1016/j.bodyim.2016.02.008. 26995158 10.1016/j.bodyim.2016.02.008

