# Supplementary material for: Multidimensional body image and self-esteem: a latent profile analysis differentiating orthorexia nervosa and exercise addiction from disordered eating
Source: BMC Psychiatry. 2026 May 25;26:425. doi: 10.1186/s12888-026-08219-2 (PMC13214407; doi:10.1186/s12888-026-08219-2)
Supplement: Supplementary file 1 — Supplementary Material 1 [file 12888_2026_8219_MOESM1_ESM.docx]

**Supplementary Material**

**Confirmatory factor analysis**

**Table S1**

CFA parameter estimates of the three-factor model.

|  | Estimate | | SE | *z*-value | *p* |
| --- | --- | --- | --- | --- | --- |
|  | Standardized | Unstandardized |  |  |  |
| ExAdd =~ |  |  |  |  |  |
| EAI-R 1 | .720 | 1.000 |  |  |  |
| EAI-R 2 | .665 | 0.971 | 0.063 | 15.538 | <.001 |
| EAI-R 3 | .640 | 0.969 | 0.069 | 14.054 | <.001 |
| EAI-R 4 | .675 | 1.050 | 0.064 | 16.375 | <.001 |
| EAI-R 5 | .922 | 1.433 | 0.081 | 17.774 | <.001 |
| EAI-R 6 | .718 | 1.092 | 0.067 | 16.216 | <.001 |
| OrNe =~ |  |  |  |  |  |
| DOS 1 | .481 | 1.000 |  |  |  |
| DOS 2 | .597 | 1.548 | 0.127 | 12.216 | <.001 |
| DOS 3 | .620 | 1.415 | 0.105 | 13.444 | <.001 |
| DOS 4 | .667 | 1.428 | 0.131 | 10.939 | <.001 |
| DOS 5 | .407 | 0.959 | 0.107 | 8.940 | <.001 |
| DOS 6 | .804 | 2.016 | 0.174 | 11.579 | <.001 |
| DOS 7 | .533 | 0.889 | 0.100 | 8.880 | <.001 |
| DOS 8 | .789 | 1.938 | 0.172 | 11.245 | <.001 |
| DOS 9 | .614 | 1.557 | 0.135 | 11.563 | <.001 |
| DOS 10 | .813 | 2.108 | 0.179 | 11.807 | <.001 |
| ED =~ |  |  |  |  |  |
| EDE-Q8 1 | .809 | 1.000 |  |  |  |
| EDE-Q8 2 | .764 | 0.918 | 0.031 | 29.418 | <.001 |
| EDE-Q8 3 | .772 | 0.770 | 0.044 | 17.498 | <.001 |
| EDE-Q8 4 | .799 | 0.996 | 0.037 | 26.907 | <.001 |
| EDE-Q8 5 | .840 | 1.072 | 0.038 | 28.226 | <.001 |
| EDE-Q8 6 | .881 | 0.842 | 0.035 | 24.169 | <.001 |
| EDE-Q8 7 | .717 | 0.885 | 0.041 | 21.483 | <.001 |
| EDE-Q8 8 | .677 | 0.789 | 0.041 | 19.259 | <.001 |
| Covariances: | | | | | |
| ExAdd~~ |  |  |  |  |  |
| OrNe | .495 | 0.192 | 0.027 | 7.147 | <.001 |
| ED | .204 | 0.362 | 0.086 | 4.190 | <.001 |
| OrNe~~ |  |  |  |  |  |
| ED | .616 | 0.415 | 0.047 | 8.801 | <.001 |
| Variances: | | | | | |
| .EAI-R 1 | .481 | 0.949 | 0.078 | 12.190 | <.001 |
| .EAI-R 2 | .558 | 1.218 | 0.103 | 11.834 | <.001 |
| .EAI-R 3 | .590 | 1.387 | 0.092 | 15.103 | <.001 |
| .EAI-R 4 | .544 | 1.349 | 0.091 | 14.848 | <.001 |
| .EAI-R 5 | .149 | 0.369 | 0.101 | 3.671 | <.001 |
| .EAI-R 6 | .484 | 1.147 | 0.091 | 12.601 | <.001 |
| .DOS 1 | .769 | 0.488 | 0.028 | 17.276 | <.001 |
| .DOS 2 | .644 | 0.635 | 0.036 | 17.537 | <.001 |
| .DOS 3 | .616 | 0.471 | 0.031 | 15.113 | <.001 |
| .DOS 4 | .555 | 0.373 | 0.031 | 12.142 | <.001 |
| .DOS 5 | .835 | 0.681 | 0.036 | 19.076 | <.001 |
| .DOS 6 | .354 | 0.327 | 0.029 | 11.456 | <.001 |
| .DOS 7 | .716 | 0.292 | 0.028 | 10.569 | <.001 |
| .DOS 8 | .377 | 0.334 | 0.029 | 11.343 | <.001 |
| .DOS 9 | .622 | 0.586 | 0.043 | 13.623 | <.001 |
| .DOS 10 | .339 | 0.334 | 0.032 | 10.322 | <.001 |
| .EDE-Q8 1 | .345 | 1.629 | 0.135 | 12.030 | <.001 |
| .EDE-Q8 2 | .416 | 1.854 | 0.166 | 11.142 | <.001 |
| .EDE-Q8 3 | .404 | 1.238 | 0.105 | 11.838 | <.001 |
| .EDE-Q8 4 | .362 | 1.739 | 0.143 | 12.167 | <.001 |
| .EDE-Q8 5 | .294 | 1.481 | 0.136 | 10.911 | <.001 |
| .EDE-Q8 6 | .225 | 0.634 | 0.069 | 9.185 | <.001 |
| .EDE-Q8 7 | .486 | 2.286 | 0.169 | 13.531 | <.001 |
| .EDE-Q8 8 | .542 | 2.276 | 0.156 | 14.627 | <.001 |
| ExAdd | 1.000 | 1.024 | 0.105 | 9.754 | <.001 |
| OrNe | 1.000 | 0.147 | 0.024 | 6.188 | <.001 |
| ED | 1.000 | 3.088 | 0.190 | 16.245 | <.001 |

*Note.* ExAdd = Factor of EAI-R items, OrNe = Factor of DOS items, ED = Factor of EDE-Q8 items, EDE-Q8 = Short Eating Disorder Examination-Questionnaire, DOS = Düsseldorf Orthorexia Scale, EAI-R = Revised Exercise Addiction Inventory.

**Table S2**

Modification indices ≥ of the CFA three-factor model.

| Parameter | | | MI | Expected Parameter Change | |
| --- | --- | --- | --- | --- | --- |
|  |  |  |  | Unstandardized | Standardized |
| ED | =~ | DOS 5 | 105.107 | 0.180 | .350 |
| OrNe | =~ | EDE-Q8 3 | 98.453 | -1.947 | -.426 |
| ED | =~ | EAI-R 5 | 88.597 | -0.196 | -.218 |
| ED | =~ | DOS 10 | 78.122 | -0.197 | -.348 |
| OrNe | =~ | EAI-R 5 | 77.809 | -1.161 | -.283 |
| ED | =~ | DOS 6 | 72.982 | -0.185 | -.339 |
| OrNe | =~ | EDE-Q8 1 | 69.897 | -1.767 | -.311 |
| ED | =~ | DOS 1 | 63.901 | 0.130 | .286 |
| OrNe | =~ | EDE-Q8 4 | 63.514 | 1.698 | .297 |
| ExAdd | =~ | DOS 5 | 63.300 | -0.219 | -.245 |
| ExAdd | =~ | EDE-Q8 1 | 62.783 | -0.422 | -.197 |
| ExAdd | =~ | EDE-Q8 3 | 62.462 | -0.366 | -.212 |
| OrNe | =~ | EDE-Q8 7 | 59.726 | 1.569 | .277 |
| ExAdd | =~ | EDE-Q8 4 | 54.086 | 0.398 | .184 |
| EDE-Q8 7 | ~~ | EDE-Q8 8 | 51.229 | -1.327 | -.582 |
| OrNe | =~ | EDE-Q8 8 | 49.959 | 1.360 | .254 |
| ExAdd | =~ | EDE-Q8 7 | 49.624 | 0.363 | .169 |
| OrNe | =~ | EDE-Q8 2 | 44.102 | -1.371 | -.249 |
| ExAdd | =~ | EDE-Q8 8 | 42.798 | 0.321 | .158 |
| ExAdd | =~ | DOS 1 | 40.910 | -0.160 | -.204 |
| OrNe | =~ | EAI-R 2 | 36.766 | -0.682 | -.177 |
| EDE-Q8 4 | ~~ | EDE-Q8 5 | 36.550 | -1.296 | -.808 |
| OrNe | =~ | EDE-Q8 5 | 36.450 | 1.348 | .230 |
| OrNe | =~ | EAI-R 3 | 35.494 | 0.648 | .162 |
| DOS 1 | ~~ | DOS 5 | 35.432 | -0.187 | -.324 |
| ED | =~ | EAI-R 3 | 33.682 | 0.103 | .119 |
| EDE-Q8 4 | ~~ | EDE-Q8 8 | 33.588 | -1.113 | -.560 |
| ExAdd | =~ | EDE-Q8 2 | 31.938 | -0.292 | -.140 |
| ED | =~ | EAI-R 2 | 30.570 | -0.099 | -.118 |
| EDE-Q8 4 | ~~ | EDE-Q8 7 | 29.699 | -1.094 | -.549 |
| DOS 5 | ~~ | EDE-Q8 4 | 27.562 | 0.431 | .396 |
| EAI-R 5 | ~~ | EDE-Q8 3 | 26.059 | -0.642 | -.950 |
| ED | =~ | EAI-R 1 | 25.718 | 0.086 | .108 |
| ExAdd | =~ | DOS 6 | 24.638 | 0.161 | .170 |
| ExAdd | =~ | DOS 10 | 24.389 | 0.166 | .169 |
| OrNe | =~ | EAI-R 4 | 23.624 | 0.565 | .137 |
| ExAdd | =~ | EDE-Q8 5 | 23.524 | 0.271 | .122 |
| DOS 1 | ~~ | EDE-Q8 7 | 23.147 | 0.352 | .333 |
| ED | =~ | DOS 3 | 22.219 | 0.089 | .180 |
| EDE-Q8 1 | ~~ | EDE-Q8 2 | 21.753 | -0.951 | -.547 |
| EAI-R 5 | ~~ | EDE-Q8 1 | 21.536 | -0.647 | -.834 |
| EDE-Q8 5 | ~~ | EDE-Q8 7 | 21.459 | -0.965 | -.524 |
| ED | =~ | EAI-R 4 | 21.341 | 0.087 | .097 |
| DOS 1 | ~~ | EDE-Q8 8 | 20.774 | 0.31 | .294 |
| DOS 10 | ~~ | EDE-Q8 6 | 20.421 | -0.358 | -.778 |
| DOS 5 | ~~ | EDE-Q8 7 | 20.385 | 0.370 | .296 |
| OrNe | =~ | EDE-Q8 6 | 20.221 | -0.813 | -.185 |
| DOS 5 | ~~ | EDE-Q8 5 | 19.823 | 0.371 | .370 |
| DOS 2 | ~~ | DOS 9 | 19.565 | -0.173 | -.284 |
| DOS 1 | ~~ | EDE-Q8 4 | 19.517 | 0.325 | .353 |
| EAI-R 1 | ~~ | EDE-Q8 4 | 18.919 | 0.546 | .425 |
| DOS 5 | ~~ | EDE-Q8 8 | 18.820 | 0.326 | .262 |
| DOS 8 | ~~ | EDE-Q8 3 | 17.998 | -0.429 | -.668 |
| DOS 6 | ~~ | EDE-Q8 6 | 17.758 | -0.334 | -.735 |
| EAI-R 5 | ~~ | EDE-Q8 6 | 16.984 | -0.475 | -.982 |
| OrNe | =~ | EAI-R 1 | 16.087 | 0.428 | .117 |
| ExAdd | =~ | EDE-Q8 6 | 15.476 | -0.175 | -.105 |
| DOS 10 | ~~ | EDE-Q8 3 | 14.305 | -0.352 | -.548 |
| DOS 1 | ~~ | DOS 2 | 14.297 | -0.122 | -.219 |
| EAI-R 2 | ~~ | EDE-Q8 3 | 14.034 | -0.478 | -.389 |
| EAI-R 6 | ~~ | DOS 5 | 13.967 | -0.214 | -.242 |
| DOS 1 | ~~ | DOS 3 | 13.957 | -0.119 | -.248 |
| EAI-R 1 | ~~ | EDE-Q8 8 | 13.741 | 0.437 | .297 |
| EAI-R 3 | ~~ | EDE-Q8 7 | 13.600 | 0.489 | .275 |
| EAI-R 3 | ~~ | EAI-R 4 | 13.551 | -0.412 | -.302 |
| EAI-R 1 | ~~ | EDE-Q8 7 | 13.463 | 0.449 | .305 |
| EDE-Q8 5 | ~~ | EDE-Q8 8 | 13.271 | -0.730 | -.398 |
| EAI-R 4 | ~~ | EDE-Q8 4 | 13.189 | 0.519 | .339 |
| DOS 3 | ~~ | EDE-Q8 4 | 13.050 | 0.301 | .333 |
| EAI-R 3 | ~~ | EDE-Q8 4 | 12.958 | 0.507 | .326 |
| DOS 2 | ~~ | EDE-Q8 8 | 12.834 | 0.304 | .253 |
| EDE-Q8 2 | ~~ | EDE-Q8 8 | 12.756 | 0.68 | .331 |
| DOS 3 | ~~ | EDE-Q8 8 | 12.296 | 0.264 | .255 |
| EAI-R 4 | ~~ | EDE-Q8 8 | 12.123 | 0.465 | .265 |
| ExAdd | =~ | DOS 3 | 12.035 | -0.100 | -.116 |
| EAI-R 5 | ~~ | EDE-Q8 2 | 11.802 | -0.476 | -.576 |
| DOS 5 | ~~ | EDE-Q8 6 | 11.749 | 0.225 | .342 |
| EAI-R 4 | ~~ | EAI-R 6 | 11.728 | -0.382 | -.307 |
| DOS 2 | ~~ | DOS 5 | 11.643 | -0.125 | -.190 |
| DOS 1 | ~~ | EDE-Q8 5 | 11.628 | 0.258 | .303 |
| EAI-R 1 | ~~ | DOS 5 | 11.052 | -0.170 | -.212 |
| EDE-Q8 1 | ~~ | EDE-Q8 8 | 10.979 | 0.647 | .336 |
| ED | =~ | DOS 9 | 10.764 | 0.068 | .123 |
| EAI-R 3 | ~~ | EAI-R 6 | 10.657 | -0.362 | -.287 |
| EAI-R 4 | ~~ | EDE-Q8 7 | 10.456 | 0.444 | .253 |
| EAI-R 5 | ~~ | EAI-R 6 | 10.261 | 0.387 | .595 |
| DOS 6 | ~~ | EDE-Q8 3 | 10.243 | -0.288 | -.453 |
| DOS 9 | ~~ | EDE-Q8 5 | 10.127 | 0.307 | .329 |
| DOS 1 | ~~ | DOS 10 | 10.121 | 0.115 | .284 |

*Note.* ExAdd = Factor of EAI-R items, OrNe = Factor of DOS items, ED = Factor of EDE-Q8 items, EDE-Q8 = Short Eating Disorder Examination-Questionnaire, DOS = Düsseldorf Orthorexia Scale, EAI-R = Revised Exercise Addiction Inventory.

**Exploratory factor analysis**

**Figure S1**

Scree-plot of the EFA for principal component analysis (PC) and factor analysis (FA).


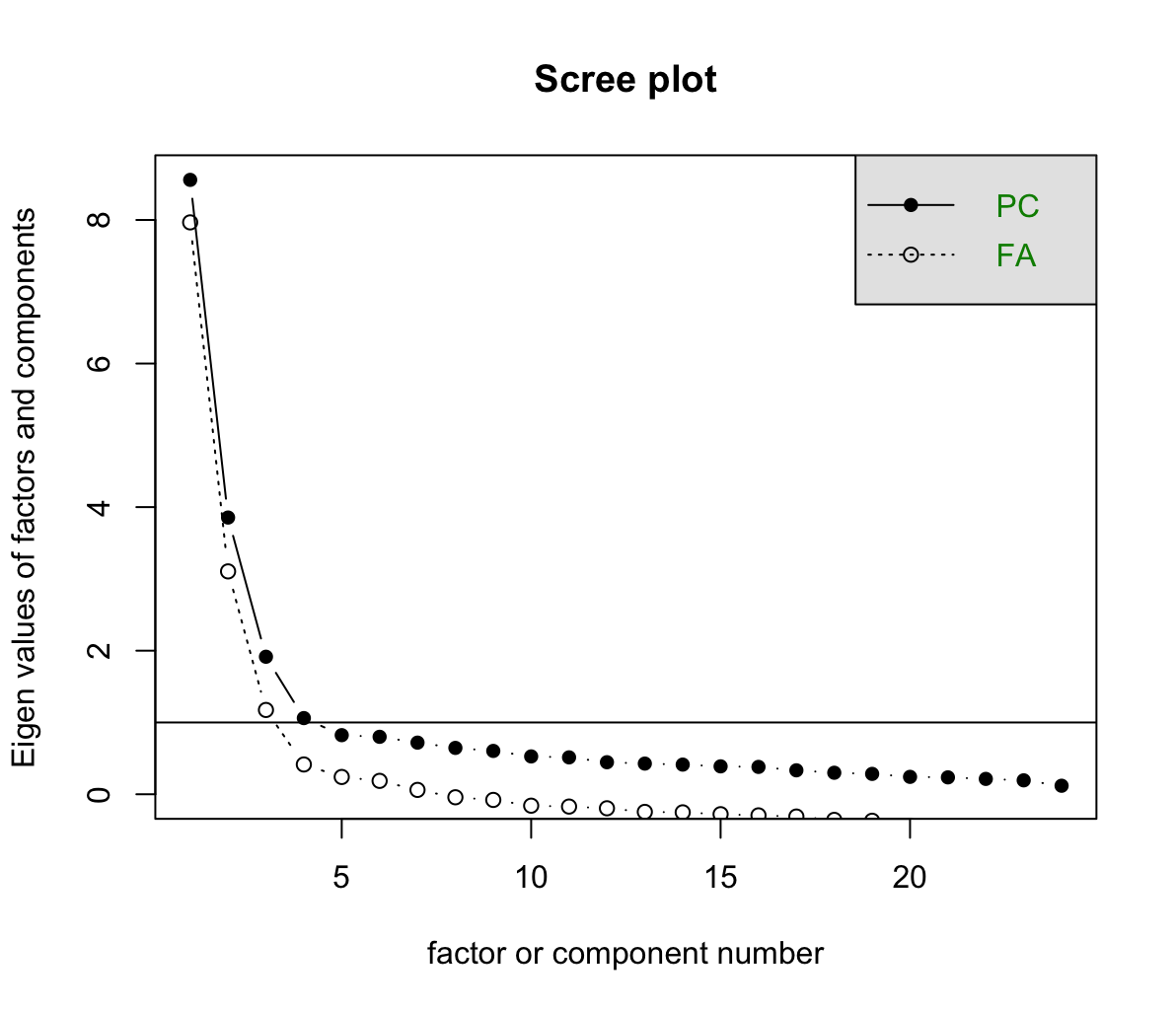


**Table S3**

EFA loading WLS-estimates of the four-factor solution after oblimin rotation, with Communality h^2^, Uniqueness u^2^, and Factor Complexity.

|  | Loadings | | | | h^2^ | u^2^ | Complexity |
| --- | --- | --- | --- | --- | --- | --- | --- |
|  | Factor 1 | Factor 3 | Factor 2 | Factor 4 |  |  |  |
| EAI-R 1 | -.11 | .05 | .76 | -.04 | .60 | .40 | 1.1 |
| EAI-R 2 | .00 | .20 | .52 | -.12 | .38 | **.62** | 1.4 |
| EAI-R 3 | -.03 | -.12 | .78 | .04 | .56 | .44 | 1.1 |
| EAI-R 4 | -.03 | -.08 | .78 | .04 | .57 | .43 | 1.0 |
| EAI-R 5 | .12 | .11 | .76 | -.10 | .68 | .32 | 1.1 |
| EAI-R 6 | .01 | -.05 | .76 | .06 | .57 | .43 | 1.0 |
| DOS 1 | -.15 | .47 | .11 | .33 | .42 | **.58** | 2.2 |
| DOS 2 | .01 | .51 | .04 | .30 | .43 | **.57** | 1.6 |
| DOS 3 | -.05 | .60 | .09 | .11 | .43 | **.57** | 1.1 |
| DOS 4 | .01 | .73 | -.02 | -.03 | .52 | .48 | 1.0 |
| DOS 5 | -.18 | .41 | .14 | .28 | .33 | **.67** | 2.5 |
| DOS 6 | .33 | .53 | .06 | -.04 | .58 | .42 | 1.7 |
| DOS 7 | -.03 | .66 | .01 | -.19 | .41 | .59 | 1.2 |
| DOS 8 | .15 | .64 | .09 | .05 | .59 | .41 | 1.2 |
| DOS 9 | -.08 | .72 | -.04 | .07 | .48 | .52 | 1.1 |
| DOS 10 | .34 | .54 | .07 | -.06 | .59 | .41 | 1.7 |
| EDE-Q 1 | .62 | .13 | .10 | .34 | .66 | .34 | 1.7 |
| EDE-Q 2 | .58 | .14 | .05 | .37 | .61 | .39 | 1.8 |
| EDE-Q 3 | .42 | .46 | .06 | -.11 | .58 | .42 | 2.1 |
| EDE-Q 4 | .90 | -.03 | -.04 | -.03 | .78 | .22 | 1.0 |
| EDE-Q 5 | .90 | -.04 | .00 | .06 | .78 | .22 | 1.0 |
| EDE-Q 6 | .71 | .23 | .08 | -.09 | .74 | .26 | 1.3 |
| EDE-Q 7 | .84 | -.06 | -.03 | -.02 | .67 | .33 | 1.0 |
| EDE-Q 8 | .79 | -.01 | -.03 | -.15 | .63 | .37 | 1.1 |

*Note.* EDE-Q8 = Short Eating Disorder Examination-Questionnaire, DOS = Düsseldorf Orthorexia Scale, EAI-R = Revised Exercise Addiction Inventory. Sufficient loadings ≥ .40 are highlighted in grey. High uniqueness ≥ .50 in bold.

**Table S4**

EFA summary of factor extraction and factor correlations of the four-factor solution after oblimin rotation.

|  | Factor 1 | Factor 3 | Factor 2 | Factor 4 |
| --- | --- | --- | --- | --- |
| Sum of Squared Loadings | 4.98 | 4.37 | 3.49 | 0.76 |
| Proportion of Variance | 0.21 | 0.18 | 0.15 | 0.03 |
| Cumulative Variance | 0.21 | 0.39 | 0.54 | 0.57 |
| Proportion Explained | 0.37 | 0.32 | 0.26 | 0.06 |
| Cumulative Proportion | 0.37 | 0.69 | 0.94 | 1.00 |
| Correlations | | | | |
| Factor 1 | 1.00 |  |  |  |
| Factor 3 | 0.45 | 1.00 |  |  |
| Factor 2 | 0.10 | 0.42 | 1.00 |  |
| Factor 4 | 0.01 | 0.20 | 0.20 | 1.00 |

**Table S5**

EFA loading WLS-estimates of the three-factor solution after oblimin-rotation, with Communality h^2^, Uniqueness u^2^, and Factor Complexity.

|  | Loadings | | | h^2^ | u^2^ | Complexity |
| --- | --- | --- | --- | --- | --- | --- |
|  | Factor 1 | Factor 2 | Factor 3 |  |  |  |
| EAI-R 1 | -.09 | .04 | 0.75 | .59 | .41 | 1.0 |
| EAI-R 2 | .03 | .15 | 0.50 | .35 | **.65** | 1.2 |
| EAI-R 3 | -.03 | -.10 | 0.78 | .56 | .44 | 1.0 |
| EAI-R 4 | -.03 | -.06 | 0.78 | .57 | .43 | 1.0 |
| EAI-R 5 | .15 | .08 | 0.75 | .66 | .34 | 1.1 |
| EAI-R 6 | .00 | -.02 | 0.76 | .57 | .43 | 1.0 |
| DOS 1 | -.18 | .58 | 0.12 | .35 | **.65** | 1.3 |
| DOS 2 | -.04 | .62 | 0.04 | .39 | **.61** | 1.0 |
| DOS 3 | -.06 | .65 | 0.08 | .44 | **.56** | 1.0 |
| DOS 4 | .04 | .71 | -0.04 | .50 | **.50** | 1.0 |
| DOS 5 | -.21 | .51 | 0.14 | .29 | **.71** | 1.5 |
| DOS 6 | .35 | .52 | 0.04 | .57 | .43 | 1.7 |
| DOS 7 | .03 | .55 | -0.01 | .32 | **.68** | 1.0 |
| DOS 8 | .15 | .66 | 0.07 | .59 | .41 | 1.1 |
| DOS 9 | -.08 | .75 | -0.06 | .48 | .52 | 1.0 |
| DOS 10 | .35 | .52 | 0.05 | .58 | .42 | 1.8 |
| EDE-Q 1 | .54 | .28 | 0.11 | .55 | .45 | 1.6 |
| EDE-Q 2 | .50 | .29 | 0.07 | .48 | .52 | 1.7 |
| EDE-Q 3 | .44 | .42 | 0.04 | .56 | .44 | 2.0 |
| EDE-Q 4 | .93 | -.05 | -0.03 | .82 | .18 | 1.0 |
| EDE-Q 5 | .91 | -.02 | 0.02 | .81 | .19 | 1.0 |
| EDE-Q 6 | .74 | .20 | 0.07 | .74 | .26 | 1.2 |
| EDE-Q 7 | .85 | -.07 | -0.03 | .67 | .33 | 1.0 |
| EDE-Q 8 | .81 | -.07 | -0.03 | .62 | .38 | 1.0 |

*Note.* EDE-Q8 = Short Eating Disorder Examination-Questionnaire, DOS = Düsseldorf Orthorexia Scale, EAI-R = Revised Exercise Addiction Inventory. Sufficient loadings ≥ .40 are highlighted in grey. High uniqueness ≥ .50 in bold.

**Table S6**

EFA summary of factor extraction and factor correlations of the three-factor solution after oblimin rotation.

|  | Factor 1 | Factor 3 | Factor 2 |
| --- | --- | --- | --- |
| Sum of Squared Loadings | 5.02 | 4.61 | 3.43 |
| Proportion of Variance | 0.21 | 0.19 | 0.14 |
| Cumulative Variance | 0.21 | 0.40 | 0.54 |
| Proportion Explained | 0.38 | 0.35 | 0.26 |
| Cumulative Proportion | 0.38 | 0.74 | 1.00 |
| Correlations | | | |
| Factor 1 | 1.00 |  |  |
| Factor 3 | 0.43 | 1.00 |  |
| Factor 2 | 0.09 | 0.44 | 1.00 |

**Table S7**

Mean differences and 95%-Confidence Intervals of post hoc latent profile comparisons.

|  | DisOrEx vs. | | | | DisEat vs. | | | AddEx vs. | | NonPath vs. LowCom |
| --- | --- | --- | --- | --- | --- | --- | --- | --- | --- | --- |
|  | DisEat | AddEx | NonPath | LowCom | AddEx | NonPath | LowCom | NonPath | LowCom |  |
| EDE-Q8 | **-0.9**  **(-1.26, -0.54)** | **-3.1**  **(-3.4, -2.79)** | **-4.12**  **(-4.42, -3.83)** | **-3.27**  **(-3.67, -2.87)** | **-2.2**  **(-2.44, -1.96)** | **-3.22**  **(-3.45, -3.00)** | **-2.37**  **(-2.73, -2.01)** | **-1.03**  **(-1.14, -0.91)** | -0.17  (-0.48, 0.13) | **0.85**  **(0.56, 1.14)** |
| DOS | **-11.58**  **(-13.03, -10.14)** | **-9.88**  **(-11.26, -8.51)** | **-14.17**  **(-15.5, -12.84)** | **-17.45**  **(-18.8, -16.11)** | **1.7**  **(0.57, 2.83)** | **-2.59**  **(-3.67, -1.51)** | **-5.87**  **(-6.97, -4.77)** | **-4.29**  **(-5.27, -3.31)** | **-7.57**  **(-8.57, -6.57)** | **-3.28**  **(-4.22, -2.34)** |
| EAI-R | **-9.07**  **(-11.42, -6.72)** | **-4.24**  **(-6.39, -2.1)** | **-7.47**  **(-9.79, -5.15)** | **-16.61**  **(-18.67, -14.55)** | **4.83**  **(3.11, 6.55)** | 1.6  (-0.33, 3.54) | **-7.54**  **(-9.15, -5.93)** | **-3.22**  **(-4.9, -1.55)** | **-12.36**  **(-13.65, -11.08)** | **-9.14**  **(-10.7, -7.57)** |
| AO | -0.19  (-0.42, 0.03) | **-0.36**  **(-0.56, -0.15)** | **-0.64**  **(-0.86, -0.42)** | **-0.73**  **(-1.03, -0.43)** | -0.16  (-0.35, 0.02) | **-0.45**  **(-0.66, -0.24)** | **-0.54**  **(-0.83, -0.25)** | **-0.29**  **(-0.47, -0.1)** | **-0.37**  **(-0.65, -0.1)** | -0.09  (-0.38, 0.2) |
| AE | 0.31  (0.00, 0.62) | **1.13**  **(0.86, 1.40)** | **1.3**  **(1.02, 1.58)** | **0.68**  **(0.3, 1.07)** | **0.82**  **(0.61, 1.04)** | **1.00**  **(0.76, 1.23)** | **0.38**  **(0.03, 0.73)** | 0.17  (0.00, 0.34) | **-0.45**  **(-0.76, -0.13)** | **-0.62**  **(-0.94, -0.29)** |
| OWP | **-0.74**  **(-1.02, -0.46)** | **-1.62**  **(-1.87, -1.37)** | **-2.33**  **(-2.57, -2.08)** | **-2.16**  **(-2.5, -1.82)** | **-0.88**  **(-1.11, -0.65)** | **-1.59**  **(-1.81, -1.36)** | **-1.42**  **(-1.75, -1.1)** | **-0.71**  **(-0.88, -0.53)** | **-0.54**  **(-0.84, -0.25)** | 0.16  (-0.13, 0.46) |
| SCW | **0.41**  **(0.16, 0.66)** | -0.11  (-0.33, 0.1) | **-0.26**  **(-0.48, -0.05)** | 0.07  (-0.24, 0.39) | **-0.52**  **(-0.70, -0.34)** | **-0.67**  **(-0.85, -0.49)** | **-0.33**  **(-0.63, -0.04)** | **-0.15**  **(-0.28, -0.03)** | 0.18  (-0.08, 0.45) | **0.34**  **(0.07, 0.6)** |
| FO | **-0.71**  **(-0.98, -0.44)** | 0.07  (-0.17, 0.32) | -0.16  (-0.43, 0.1) | **-1.3**  **(-1.66, -0.94)** | **0.79**  **(0.56, 1.01)** | **0.55**  **(0.3, 0.8)** | **-0.59**  **(-0.94, -0.24)** | **-0.24**  **(-0.45, -0.02)** | **-1.37**  **(-1.7, -1.04)** | **-1.14**  **(-1.48, -0.79)** |
| FE | -0.24  (-0.52, 0.05) | **0.37**  **(0.14, 0.61)** | **0.39**  **(0.13, 0.65)** | -0.20  (-0.60, 0.21) | **0.61**  **(0.38, 0.84)** | **0.63**  **(0.38, 0.88)** | 0.04  (-0.36, 0.44) | 0.02  (-0.18, 0.21) | **-0.57**  **(-0.94, -0.2)** | **-0.59**  **(-0.96, -0.21)** |
| HO | **-0.56**  **(-0.76, -0.35)** | -0.10  (-0.28, 0.09) | **-0.27**  **(-0.48, -0.07)** | **-0.97**  **(-1.24, -0.69)** | **0.46**  **(0.29, 0.63)** | **0.28**  **(0.09, 0.47)** | **-0.41**  **(-0.68, -0.14)** | **-0.18**  **(-0.34, -0.01)** | **-0.87**  **(-1.12, -0.62)** | **-0.69**  **(-0.96, -0.43)** |
| IO | -0.12  (-0.39, 0.15) | -0.07  (-0.31, 0.18) | -0.08  (-0.34, 0.19) | **-0.67**  **(-1.03, -0.31)** | 0.05  (-0.17, 0.28) | 0.04  (-0.2, 0.29) | **-0.55**  **(-0.9, -0.2)** | -0.01  (-0.23, 0.21) | **-0.61**  **(-0.94, -0.28)** | **-0.6**  **(-0.94, -0.25)** |
| HE | 0.15  (-0.14, 0.45) | **0.55**  **(0.29, 0.8)** | **0.62**  **(0.35, 0.89)** | **0.47**  **(0.11, 0.83)** | **0.39**  **(0.17, 0.62)** | **0.46**  **(0.22, 0.71)** | 0.32  (-0.02, 0.65) | 0.07  (-0.12, 0.26) | -0.08  (-0.38, 0.23) | -0.15  (-0.46, 0.17) |
| RSES | **4.00**  **(1.40, 6.61)** | **8.20**  **(6.04, 10.35)** | **9.81**  **(7.55, 12.08)** | **7.51**  **(4.27, 10.76)** | **4.2**  **(2.20, 6.19)** | **5.81**  **(3.70, 7.92)** | **3.51**  **(0.37, 6.65)** | **1.62**  **(0.11, 3.12)** | -0.69  (-3.48, 2.11) | -2.30  (-5.18, 0.58) |

*Note.* EDE-Q8 = Short Eating Disorder Examination-Questionnaire, DOS = Düsseldorf Orthorexia Scale, EAI-R = Revised Exercise Addiction Inventory, AO = appearance orientation, AE = appearance evaluation, OWP = overweight preoccupation, SCW = self-classified weight, FO = fitness orientation, FE = fitness evaluation, HO = health orientation, IO = illness orientation, HE = health orientation, RSES = Rosenberg Self-Esteem Scale. Bold values indicate significant mean differences at *p* < .05.
